# Update on the EFFECTS study of fluoxetine for stroke recovery: a randomised controlled trial in Sweden

**DOI:** 10.1186/s13063-020-4124-7

**Published:** 2020-02-28

**Authors:** Erik Lundström, Eva Isaksson, Per Näsman, Per Wester, Björn Mårtensson, Bo Norrving, Håkan Wallén, Jörgen Borg, Martin Dennis, Gillian Mead, Graeme J. Hankey, Maree L. Hackett, Katharina S. Sunnerhagen, For the EFFECTS Trial Collaboration, Ann Charlotte Laska, Bjarni Gudmundsson, Björn Cederin, Magnus Esbjörnsson, Bernice Wiberg, Maria Lantz, Jörg Teichert, Magnus Bergmann, Lennart Welin, Ulrika Löfmark, Sven-Erik Bysell, Xiaolei Hu, Axel Andersson, Moa Gunnarsson, Pernilla Sandgren, Eva Ask, Peter Thomasson Sommer, Bo Danielsson, Liisa Hopia, Andreas Arvidsson, Fredrik Björck, Anke Brederlau, Trandur Ulfarsson, Mehran Taklif, Magnus Gibson, Brita Eklund, Björn Hedström, Ellinore Richardsson, Daniel Merrick, Per Broman, Max Mademyr-Larsson, Andreas Ranhem, Camilla Ronnheden, Martin Johansson, Anette Onkenhout

**Affiliations:** 1 Department of Neuroscience, Neurology, Uppsala University, Akademiska Sjukhuset, SE-751 85 Uppsala, Sweden; 2 Department of Clinical Neuroscience, Neurology, Karolinska Institutet, Nobels väg 6, SE-171 76 Stockholm, Sweden; 3 Centre for Safety Research, KTH Royal Institute of Technology, TR10 B, SE-100 44 Stockholm, Sweden; 4 Department of Clinical Sciences, Danderyd Hospital, Karolinska Institutet, SE-182 88 Stockholm, Sweden; 5 Department of Public Health & Clinical Medicine, Umeå University, SE-901 87 Umeå, Sweden; 6 Department of Clinical Neuroscience, Karolinska Institutet, SE-171 77 Stockholm, Sweden; 7 Department of Clinical Sciences, Lund, Neurology, Skåne University Hospital, Lund University, SE-221 85 Lund, Sweden; 8 Department of Clinical Sciences, Division of Cardiovascular Medicine, Danderyd Hospital, Karolinska Institutet, SE-182 88 Stockholm, Sweden; 9 Royal Infirmary, University of Edinburgh, Edinburgh, EH16 4SB UK; 10 Medical School, The University of Western Australia, Perth, Australia; 11 The George Institute for Global Health, Faculty of Medicine, University of New South Wales Sydney, Sydney, Australia; 12 The University of Central Lancashire, Preston, UK; 13 Institute of Neuroscience and Physiology, The Sahlgrenska Academy, University of Gothenburg, Gothenburg, Sweden; 14 Riga Stradins University, Riga, Latvia; 15 Sunnaas Rehabilitation Hospital, Bjørnemy, Norway; 16 Rehabilitation Medicine, Sahlgrenska University Hospital, Gothenburg, Sweden

**Keywords:** Stroke, Fluoxetine, Selective serotonin reuptake inhibitor, SSRI, Stroke recovery, Recovery of function, Multicentre study

## Abstract

**Abstract:**

Studies have suggested that fluoxetine might improve neurological recovery after stroke, but the results remain inconclusive. The EFFECTS (Efficacy oF Fluoxetine – a randomisEd Controlled Trial in Stroke) reached its recruitment target of 1500 patients in June 2019. The purpose of this article is to present all amendments to the protocol and describe how we formed the EFFECTS trial collaboration in Sweden.

**Methods:**

In this investigator-led, multicentre, parallel-group, randomised, placebo-controlled trial, we enrolled non-depressed stroke patients aged 18 years or older between 2 and 15 days after stroke onset. The patients had a clinical diagnosis of stroke (ischaemic or intracerebral haemorrhage) with persisting focal neurological deficits. Patients were randomised to fluoxetine 20 mg or matching placebo capsules once daily for 6 months.

**Results:**

Seven amendments were made and included clarification of drug interaction between fluoxetine and metoprolol and the use of metoprolol for severe heart failure as an exclusion criterion, inclusion of data from central Swedish registries and the Swedish Stroke Register, changes in informed consent from patients, and clarification of design of some sub-studies.

EFFECTS recruited 1500 patients at 35 centres in Sweden between 20 October 2014 and 28 June 2019. We plan to unblind the data in January 2020 and report the primary outcome in May 2020.

**Conclusion:**

EFFECTS will provide data on the safety and efficacy of 6 months of treatment with fluoxetine after stroke in a Swedish health system setting. The data from EFFECTS will also contribute to an individual patient data meta-analysis.

**Trial registration:**

EudraCT 2011-006130-16. Registered on 8 August 2014.

ISRCTN, ISRCTN13020412. Registered on 19 December 2014.

ClinicalTrials.gov: NCT02683213. Retrospectively registered on 2 February 2016.

## Introduction

### Background

Globally, 13.7 million new strokes occur annually [1]. In Sweden, with a population of about 10 million inhabitants, about 23,000 people experience stroke annually [2]. Despite major improvements in the treatment of acute ischaemic stroke over the past 20 years, about half of all stroke survivors are left with long-term residual disability [3].

In 2011, the FLAME trial [4] reported promising results for the effects of fluoxetine on stroke recovery. FLAME was a randomised controlled trial (RCT) of 118 patients with ischaemic stroke and unilateral motor weakness; half of the patients were randomised to 20 mg fluoxetine and half to placebo daily for 3 months as well as receiving physiotherapy. At 3 months, the proportion of patients with a modified Rankin Scale (mRS) [5] of 0–2 was 17 absolute percent higher in the fluoxetine group (26% versus 9%, *p* = 0.015). A subsequent Cochrane review of 52 RCTs (*N* = 4059) of selective serotonin reuptake inhibitors (SSRIs) for stroke recovery [6] showed that SSRIs improved functional recovery after stroke. However, most trials were small and prone to systematic and random errors. The authors concluded that large, well-designed trials were needed to determine whether SSRIs were indeed safe and effective in improving functional outcome after stroke.

This led us to develop a family of three large trials of fluoxetine for stroke recovery [7]: EFFECTS (Efficacy oF Fluoxetine – a randomisEd Controlled Trial in Stroke), AFFINITY (The Assessment oF FluoxetINe In sTroke recovery) and FOCUS (Fluoxetine Or Control Under Supervision) [8]. Each trial was funded and run separately with oversight from its own Steering Committee. We hypothesised that the routine administration of 20 mg fluoxetine once daily in the 6 months after an acute stroke improves the patient’s functional outcome.

The FOCUS trial (*N* = 3127) is the only trial to date to have finished and published its results [8]. For the primary outcome measure, the distribution across mRS categories at 6 months was similar in the fluoxetine and placebo groups (common odds ratio adjusted for minimisation variables 0.95 [95% CI 0.84–1.08]; *p* = 0.44). Patients allocated fluoxetine were less likely than those allocated placebo to develop new depression by 6 months (13.4% vs 17.2%, *p* = 0.0033), but they had more bone fractures (2.9% vs 1.5%; *p* = 0.007).

The EFFECTS (*N* = 1500) and AFFINITY (*N* = 1280) trials finished their recruitment in June 2019 and are due to report their primary outcome at the stroke conference jointly organised by the European Stroke Organisation and the World Stroke Organization in Vienna in May 2020. Further, we plan to present an individual patient data meta-analysis from the three trials. Finally, we will combine our data and update the Cochrane systematic review of selective serotonin reuptake inhibitors (SSRIs) for stroke recovery [6].

This update follows the Standard Protocol Items: Recommendations for Interventional Trials (SPIRIT) 2013 checklist [9] in combination with the 2013 SPIRIT explanation and elaboration guidance for protocols of clinical trials [10] (Additional files 1 and 2).

The purpose of this update is to present all amendments to EFFECTS and describe how we formed the EFFECTS trial collaboration. We also describe the settings and locations in which the study was performed.

## Methods

### Design overview

EFFECTS is a multicentre, randomised, placebo-controlled, blinded, parallel group trial of fluoxetine for stroke recovery performed at 35 centres in Sweden. Primary outcome was the mRS at 6 months. Recruitment started 20 October 2014 and ended 28 June 2019, when the target of 1500 patients was met. We plan to unblind the dataset when the last patient has had their 6-month follow-up in January 2020 and report the primary outcome in May 2020.

For description of the core study protocol, including study procedures and data collection, allocation and blinding procedures, we refer to the published trial protocol publication [7] and statistical analysis plan [11].

### Important changes after trial commencement

During the course of the study, we made seven amendments to the protocol. All these amendments, the Research Ethical Committee approvals and the approvals from the Swedish Medical Product Agency are available in Additional file 3. For convenience, we have summarised the amendments and their justification in a table (Additional file 4).

The latest version of the protocol (version 5.0 28 February 2018) is available online on the study’s website [12]. All previous versions including amendments were published on the homepage and communicated to active centres during the course of the study.

Below, we list the two most important changes.

1. In Amendment 2, we changed the patient consent form. In this version, the patient permits EFFECTS to obtain information from the central Swedish registries regarding sick leave, care-related consumption of resources and survival. Registry data are more accurate when collecting health economics data than asking patients for this information [13]. In this way, the responder burden for patients was reduced.
  Amendment 2 was approved 10 June 2015. All patients who had signed the old consent form (25 April 2013, v 2), had to re-sign the new version (18 May 2015, v 3). On 25 May 2018, the General Data Protection Regulation (GDPR) was implemented. Since GDPR is a regulation governed by EU law, we had to change the consent form, which is reflected in v 4, 25 May 2018 (Additional file 5). This change, however, was not accompanied by any amendment or re-signing of consent. We informed all patients who had signed the previous consents via a personal letter and updated our homepage with the information.
2. About safety. The company that manufactured our Investigational Medicinal Product, informed us on 22 November 2016 that they had updated their Summary of Product Characteristics. EFFECTS Steering Committee and Data Monitoring Committee concluded that a serious interaction between metoprolol and fluoxetine may be clinically significant for more advanced heart failure. Consequently, we added the following exclusion criteria:
  “Fluoxetine is contra-indicated in combination with metoprolol used in cardiac failure New York Heart Association Grade III B–IV.”
  Further, we clarified that patients treated with higher doses of metoprolol (> 100 mg/day) on the indication of heart failure, early after enrolment, should be monitored clinically and with an electrocardiogram.
  For safety reasons, we carried out a review of the medical charts of patients currently on metoprolol. Our Data Management Committee did not find any indication of serious interaction after reviewing the unblinded data and advised the Chief Investigator and the Steering Committee to carry on. Amendment 5. Approval 4 January 2017.

### Settings and locations where the data were collected

EFFECTS is performed in Sweden in which the health care is tax funded and fairly equally distributed throughout the country [14]. The government establish principles and guidelines, and sets the political agenda for health and medical care. Sweden is divided into 20 regions, each responsible for the health care in its particular area. In Sweden, hospitals can be divided in three types: university hospitals, specialised non-university hospitals, and community hospitals [15]. There is acute stroke care in all these, in total, 72 hospitals.

#### Stroke in Sweden data from Riksstroke 2018

In 2018, there were 21,124 strokes registered in Riksstroke – the Swedish stroke registry [2]. With a coverage of 90% of all strokes the estimated number of strokes is 23,735.

Ischaemic stroke accounts for 86% and intracerebral haemorrhage for 14%. In 2018, 63% had mild strokes, defined as National Institutes of Health Stroke Scale (NIHSS) 0–5 points. The mean NIHSS was 6 and the median 3 points.

More men (54%) than women (46%) have a stroke. The mean age is 75 years, 73 for men and 78 for women. Sixty-four per cent of the stroke patients have high blood pressure, 29% atrial fibrillation, 23% diabetes and 14% are smokers at onset.

#### Standard care of stroke in Sweden

In 2018, 17% of all patients with ischaemic stroke were treated with reperfusion therapy; 14% with intravenous thrombolysis only, or intravenous thrombolysis in combination with thrombectomy. Reperfusion treatment has almost tripled since 2010.

The proportion of acute stroke patients treated at a stroke unit at some point during their hospital stay is high – 92%. The median length of stay in hospital is 7 days, with substantial variation between the hospitals. One reason for the variation could be different application of early supported discharge. Approximately 85% of patients are evaluated by a physical or occupational therapist, and around a third of the patients had their speech and swallow function evaluated by a speech therapist during the hospital stay.

Three out of four return to their own home after discharge. Of those one in four are judged to have no need of any rehabilitation, according to staff at the discharging hospital.

In EFFECTS, we did not give any specific instructions to health-care personnel regarding physical or other types of training, although the local centre registered, organised and individualised training for each patient. Patients received stroke rehabilitation according to their local stroke team’s routines during the treatment period.

## Results

### Building a network, training the study personnel and initiation of sites

Early in the process we decided to meet potential investigators at their hospitals face to face instead of relying on email or telephone. This decision was based on intuition rather than a review of the literature and it led to more than 100 travelling days for the chief investigator and the trial manager.

#### How we reached out to a potential centre

First, we reached out to people we have previously worked with in the International Stroke Trial 3 [16]. This was done by a brief email about the rationale behind EFFECTS, an estimate of the time commitment, and the financial compensation for participation in the study. If the centre was interested in joining the trial, the principal investigator (PI) at each centre sent in an Expression of Interest/Eligibility form and we scheduled a Site Initiation Visit (SIV) as soon as possible. The SIV was described as an information meeting and was carried out during lunchtime. All staff at the stroke unit, and where appropriate, outpatient service, were welcomed to participate in the SIV, but it was mandatory for the intended PI and the trial nurse. For the PI and trial nurse, the meeting could last from 1 to 3 hours, depending on how familiar the centre was with RCT participation.

Second, we used the Riksstroke report to identify Stroke Units with medium to high volume care. If a centre had been awarded *Stroke Unit of the Year*, or received an *Excellent Stroke Care* mention in Riksstroke, we contacted the centre, regardless of its size.

Third, we attended several stroke meetings in Sweden and one Nordic Stroke Meeting (held in Malmö, Sweden) with an EFFECTS exhibition (Table 1).

**Table 1:** Estimated time/patient required for the local centre

Item	Time
Screening	5 min
Inclusion	60 min
1 week telephone follow-up	5 min
1 month telephone follow-up	5 min
3 months follow-up (face-to-face; sometimes by telephone)	30 min
6 months follow-up, face-to-face	60 min
7 months telephone follow-up	30 min
Entering data into the electronic case report system	60 min
Answering queries	60 min
Other: We arranged training for the study personnel in study specific moments (4 h) and Good Clinical Practice (4 h). In addition, we organised 4 investigator meetings in Sweden (1 day per meeting) and 5 meetings at European Stroke Conferences.	

Finally, on two occasions, we carried out feasibility studies in which we examined whether eligible patients and interested study personnel were available.

The study personnel were not given any personal monetary compensation. The centre, however, received 5000 SEK for each included patient. There was no upper limit to how many patients the centre could include. The EFFECTS study was done in parallel with the usual health care in Sweden.

All patients were covered by the Swedish medical insurance [17].

#### Site initiation visit

##### All personnel – during a working lunch meeting (1 hour)

The following items were discussed with the sites:

- The rationale, scientific background and hypothesis. (Chief investigator, approximately 20 minutes)
- Inclusion and exclusion criteria. Follow-up. Brief introduction to randomisation and follow-up. (Trial manager, approximately 20 minutes)
- Questions and answers (All, 20 minutes)

##### PI and trial nurse(s) – extended meeting after the lunch (1–3 hours)

After the lunch, the trial manager and the chief investigator discussed the following in detail:

- Study protocol
- Procedure for informed consent
- Patient recruitment plan/screening activities/enrolment
- Facilities and study personnel
- Randomisation procedure
- Investigational Medicinal Product handling and accountability
- Essential documents
- How the Case Report Form is filled out
- Safety reporting (adverse events/serious adverse events) and procedures for collection and documentation
- Good Clinical Practice (GCP) training and curriculum vitae

Finally, the Investigator Study File was handed over and discussed. The Investigator Study File contained essential documents required according to GCP. After all essential documents were signed and sent to the co-ordinating centre the study personnel (listed in the delegation log) were given access to the randomisations system and the centre was approved as active, ready to recruit patients.

#### Time for the local centre

Table 1 illustrates the time commitment for a typical patient and their follow-ups at the local centre.

#### Organisation and training of an EFFECTS centre

At each centre, we established a delegation list and persons on that list are referred to as study personnel. The study personnel consisted of a minimum of two people: one principal investigator (an experienced stroke physician) and one responsible trial nurse (registered nurse), both trained in GCP, the trial-specific procedures and our electronic Case Report Form (eCRF).

Further co-investigators (physicians) or trial nurses (registered nurses) were added at the discretion of the local principal investigator. We had no upper limit on how many study personnel were allowed on the delegation list, but all people who performed study-specific tasks had to be trained in GCP, study-specific instruments and eCRF. The training was organised by the co-ordinating centre at Karolinska Institutet, either at the local site or at special central training days. In total, we trained over 150 study personnel in EFFECTS. A list of all centres and study personnel is available as Additional file 6.

EFFECTS recruited patients from acute stroke and rehabilitation units. Around 3% of Swedish stroke patients go to a rehabilitation unit.

The reason for recruiting from rehabilitation units was that the median length of stay in hospital in Sweden is short – especially in the Stockholm region – and since it was possible to include patients until 15 days post stroke, we thought it would be possible to include individuals at rehabilitations units.

## Discussion

EFFECTS proves that it is possible to carry out a large investigator-led RCT in a country with only 10 million inhabitants. In fact, EFFECTS is now the largest ever stroke RCT conducted in Sweden. Further, EFFECTS is the second largest RCT of fluoxetine for stroke recovery after the FOCUS trial [8].

As a family of three investigator-led RCTs, EFFECTS, AFFINITY and FOCUS provide several benefits. Together, we wrote a strong core protocol and applied for funding in our respective countries and tailored study methods to each national setting(s). EFFECTS was able to use the same randomisation system and purchase the study drug from the same provider as FOCUS, which saved months of work. However, most important of all was probably the transfer of knowledge from experienced trialist to less experienced.

Although EFFECTS succeeded in reaching its target of 1500 participants, one major limitation was that it took longer than anticipated. We believe that the lack of a stroke research network was the main culprit. While we were able to build on an old informal network from the International Stroke Trial 3 (IST-3) [18], in many cases the previous PI or trial nurse had retired or moved. Basically, we had to build up and train our own stroke research network from scratch. In the United Kingdom, where there is a centrally funded network to support trials, our sister study FOCUS proved that recruitment rates were faster.

Data from EFFECTS will test the external validity of the FOCUS trial results and increase the precision of the estimates of the efficacy and safety of fluoxetine in ischaemic and haemorrhagic stroke. The planned individual patient data meta-analysis of EFFECTS, AFFINITY and FOCUS [8], as well as a subsequent update of the Cochrane systematic review [6] will likely give us a definitive answer as to whether fluoxetine has any role to play in stroke recovery.

## Supplementary information

**Additional file 1.** SPIRIT 2013 Checklist for EFFECTS.

**Additional file 2.** WHO Trial Registration Data Set for the EFFECTS trial.

**Additional file 3.** Research Ethical Committee and the Swedish Medical Product Agency approvals for EFFECTS.

**Additional file 4.** Overview of protocol versions in EFFECTS.

**Additional file 5.** Consent to participate in EFFECTS and consent regarding handling notes and data management.

**Additional file 6.** List of centre and study personnel in EFFECTS.

**Additional file 7.** Monitoring Plan.

**Additional file 8.** Data Management Plan for EFFECTS.

**Additional file 9.** Data Review Plan (DRP) for EFFECTS.

**Additional file 10.** EFFECTS latest DMC charter.

## Data Availability

We plan to compile an anonymised trial dataset with individual participant data and co-ordinate a data dictionary with FOCUS and AFFINITY. The datasets used and/or analysed during the current study could be made available by the corresponding author in response to a reasonable request. However, according to the Swedish Secrecy Act 24:8, an interested researcher must first apply and receive approval from a Swedish REC. Written proposals will be assessed by the EFFECTS Steering Committee and a decision made about the appropriateness of the use of data. A data sharing agreement will be put in place before any data are shared.

## Abbreviations

AFFINITY: The Assessment oF FluoxetINe In sTroke recoverY (AFFINITY)
DMC: Data Monitoring Committee
eCRF: Electronic Case Report Form
EFFECTS: Efficacy oF Fluoxetine – a randomisEd Controlled Trial in Stroke
FOCUS: Fluoxetine Or Control Under Supervision
GCP: Good Clinical Practice
GDPR: General Data Protection Regulation
mRS: modified Rankin Scale
NIHSS: National Institutes of Health Stroke Scale
PI: Principal investigator
RCT: Randomised controlled trial
SIV: Site Initiation Visit
SPIRIT: Standard Protocol Items: Recommendations for Interventional Trials
SSRI: Selective serotonin reuptake inhibitor
