# Correction to: Update on the EFFECTS study of fluoxetine for stroke recovery: a randomised controlled trial in Sweden

**DOI:** 10.1186/s13063-020-04327-w

**Published:** 2020-05-07

**Authors:** Erik Lundström, Eva Isaksson, Per Näsman, Per Wester, Björn Mårtensson, Bo Norrving, Håkan Wallén, Jörgen Borg, Martin Dennis, Gillian Mead, Graeme J. Hankey, Maree L. Hackett, Katharina S. Sunnerhagen, For the EFFECTS Trial Collaboration

**Affiliations:** 1 Department of Neuroscience, Neurology, Uppsala University, Akademiska, Sjukhuset, SE-751 85 Uppsala, Sweden; 2 Department of Clinical Neuroscience, Neurology, Karolinska Institutet, Nobels väg 6, SE-171 76 Stockholm, Sweden; 3 Centre for Safety Research, KTH Royal Institute of Technology, TR10 B, SE-100 44 Stockholm, Sweden; 4 Department of Clinical Sciences, Danderyd Hospital, Karolinska Institutet, SE-182 88 Stockholm, Sweden; 5 Department of Public Health & Clinical Medicine, Umeå University, SE-901 87 Umeå, Sweden; 6 Department of Clinical Neuroscience, Karolinska Institutet, SE-171 77 Stockholm, Sweden; 7 Department of Clinical Sciences, Lund, Neurology, Skåne University Hospital, Lund University, SE-221 85 Lund, Sweden; 8 Department of Clinical Sciences, Division of Cardiovascular Medicine, Danderyd Hospital, Karolinska Institutet, SE-182 88 Stockholm, Sweden; 9 Royal Infirmary, University of Edinburgh, Edinburgh, EH16 4SB UK; 10 Medical School, The University of Western Australia, Perth, Australia; 11 The George Institute for Global Health, Faculty of Medicine, University of New South Wales Sydney, Sydney, Australia; 12 The University of Central Lancashire, Preston, UK; 13 Institute of Neuroscience and Physiology, The Sahlgrenska Academy, University of Gothenburg, Gothenburg, Sweden; 14 Riga Stradins University, Riga, Latvia; 15 Sunnaas Rehabilitation Hospital, Bjørnemy, Norway; 16 Rehabilitation Medicine, Sahlgrenska University Hospital, Gothenburg, Sweden

**Correction to: Trials (2020) 21:233**

**https://doi.org/10.1186/s13063-020-4124-7**

Following publication of the original article [1], we were notified that one of the corresponding author’s affiliations was omitted. Affiliation no. 2 is now added to for Prof. Erik Lundström.
