# Supplementary material for: Update on the EFFECTS study of fluoxetine for stroke recovery: a randomised controlled trial in Sweden
Source: Trials. 2020 Feb 28;21:233. doi: 10.1186/s13063-020-4124-7 (PMC7048055; doi:10.1186/s13063-020-4124-7)
Supplement: Supplementary file 2 — Additional file 2. WHO Trial Registration Data Set for the EFFECTS trial. [file 13063_2020_4124_MOESM2_ESM.docx]

| **Data category** | **Information** |
| --- | --- |
| **1. Primary Registry and Trial Identifying Number** | ClinicalTrials.gov: NCT02683213 |
| **2. Date of Registration in Primary Registry** | Retrospectively registered 2 February 2016. |
| **3. Secondary Identifying Numbers** | EudraCT 2011-006130-16. Registered on 8 August 2014. ISRCTN, ISRCTN13020412. Registered on 19 December 2014. |
| **4. Source(s) of Monetary or Material Support** | EFFECTS has received grants from the   - Swedish Medical Council - Swedish Brain Foundation - Swedish Heart-Lung Foundation - King Gustav V and Queen Victoria’s Foundation of Freemasons - Swedish Stroke Association (STROKE-Riksförbundet) - Swedish Society of Medicine   All funders are non-commercial, with none from industry. None of the funders nor the sponsor had any role in the design of this study and will not have any role during its execution, analyses, interpretation of the data or decision to submit results.  The dataset is saved in Karolinska Institutet’s electronic notebook. |
| **5. Primary Sponsor** | Karolinska Institutet |
| **6. Secondary Sponsor(s)** | N/A |
| **7. Contact for Public Queries** | Karolinska Institutet, Department of Clinical Sciences, Danderyds Hospital SE-182 88 Stockholm  Trial Manager: Eva Isaksson phone: +46 70 340 48 92, email: eva.isaksson@ki.se  Trial Manager Assistant: Nina Greilert, phone: +46 70-566 02 43, email: nina.greilert@ki.se |
| **8. Contact for Scientific Queries** | Chief Investigator, Associate Professor: Erik Lundström  email: [erik.lundstrom@ki.se](mailto:erik.lundstrom@ki.se) or [erik.lundstrom@neuro.uu.se](mailto:erik.lundstrom@neuro.uu.se). |
| **9. Public Title** | Efficacy of Fluoxetine - a Trial in Stroke (EFFECTS) |
| **10. Scientific title** | Establishing the Effect(s) and Safety of Fluoxetine Initiated in the Acute Phase of Stroke |
| **11. Country of Recruitment** | Sweden |
| **12. Health Condition(s) or Problem(s) Studied** | Stroke |
| **13. Intervention(s)** | Active comparator: one capsule fluoxetine 20 mg once daily for 6 months.  Placebo comparator: one matching capsule placebo once daily for 6 months. |
| **14. Key Inclusion and Exclusion Criteria** | **Inclusion criteria**   - Informed consent could only be obtained from a patient who according to the trial investigator was mentally capable of decision-making and who, after having received information and got answers to their questions, wanted to participate in the trial. - Brain imaging compatible with intra cerebral hemorrhage or ischemic stroke. - Randomisation performed between 2 and 15 days after stroke onset and by the research group at the patient's local/emergency hospital. - Persisting focal neurological deficit present at the time of randomisation severe enough to warrant treatment from the physicians and the patient's and relative's perspective.   **Exclusion criteria**   - Subarachnoidal hemorrhage except where secondary to a primary intracerebral hemorrhage. - Unlikely to be available for follow up for the next 12 months e.g. no fixed home address. - Unable to speak Swedish and no close family member available to help with follow up forms. - Other life-threatening illness (e.g. advanced cancer) that will make 12-month survival unlikely. - History of epileptic seizures. - History of allergy or contraindications to fluoxetine including: Hepatic impairment (S-ASAT/ALAT > 3 upper normal limit) and renal impairment (S-Creatinine levels > 180 micromol/L). - Pregnant or breastfeeding, women of childbearing age not taking contraception. Minimum contraception is an oral contraceptive. An HCG-test is to be made prior randomization and after the end of trial medication. - Previous drug overdose or attempted suicide. - Already enrolled into a CTIMP. - Current or recent (within the last month) depression requiring treatment with an SSRI antidepressant.   Current use of medications which have serious interactions with fluoxetine Use of any mono-amino-oxidase inhibitor (MAOI) during the last 5 weeks. Co-administration of Fluoxetine and a mono-amino-oxidase inhibitor (MAOI) may result in life threatening interactions. Therefore, patients on MAOI are ineligible for the EFFECTS trial. Also, any patient in need of treatment with a MAOI must stop their trial treatment for at least 5 weeks before commencing the MAOI, or to be treated as in-patients by a psychiatrist. |
| **15. Study Type** | - Interventional (Clinical Trial) - Allocation: randomised - Intervention model: parallel assignment - Blinding: Quadruple (Participant, Care Provider, Investigator, Outcomes Assessor) - Phase III - We used the same randomisation system as our sister trial FOCUS (hosted by the University of Edinburgh, UK) although we translated the questions into Swedish. After obtained consent, the randomising person entered baseline data into a secure web-based randomisation system. The system checked baseline data for completeness and consistency and allocated the patient a unique EFFECTS trial patient ID and a treatment number. Patients were randomised in a 1:1 ratio to either fluoxetine or placebo. Following randomisation, the system generated an email to the main center that a patient was randomised, the name of the randomising person, center name, EFFECTS trial patient ID and treatment number which corresponded to either fluoxetine or placebo.   The system applied a minimization program to achieve balance for four factors:   1. Delay since stroke onset (2 – 8 versus 9 – 15 days) 2. Predicted 6 months outcome based on the six simple variable model 3. Presence of a motor deficit based on NIHSS at inclusion 4. Presence of aphasia based on NIHSS at inclusion   Data from the server in Edinburgh was automatically sent to a secure server in Sweden and was immediately available in our electronic case report system, OpenClinica  *The six simple variable model*  The six simple model includes six variables, four at the onset and two prior to the stroke. The four variables at the onset were: age; ability to walk unassisted; ability to talk; and whether confusion is present or not. The two variables before stroke were whether the patient was independent and living alone.  The placebo capsule was visually identical to the fluoxetine capsules, even when broken open. Patients, their families, the health-care personnel, staff in the coordinating centre – all were masked to treatment allocation. |
| **16. Date of First Enrollment** | 20 October 2014. |
| **17. Target sample Size** | 1,500 participants. |
| **18. Recruitment Status** | Last patient was recruited 28 June 2019. |
| **19. Primary Outcome(s)** | Functional status, measured with the modified Rankin scale at 6 months |
| **20. Key Secondary Outcomes** | - Survival (via central Swedish registry), at least 3 years - Modified Rankin Scale at 12 months - Health status measured with the Stroke Impact Scale (6 and 12 months) - Adverse events (6 months) - Depression using the Montgomery-Åsberg Depression Rating Scale (6 months) - Number of patients with epileptic seizures, hyponatremia, upper gastrointestinal bleeding, other major bleeding, poorly controlled diabetes, falls resulting in fractures, falls resulting in injury, new fractures. 6 and 12 months. - Fatigue measured with the vitality subscale of the Health Questionnaire (6 and 12 months) - Health-related quality of life measured with the five-level Euroqol 5D (EQ5D-5 L) (6 and 12 months) - Cost-effectiveness and cost-utility assessed by measuring costs, survival and health related quality of life (EQ5D) 6 and 12 months |
| **21. Ethics Review** | The study was approved by the Research Ethical Committee (REC) in Stockholm, Sweden on 30th September 2013. Number: 2013/1265-31/2. All the subsequent Amendments (1 – 7) were approved by the same REC.   1. Amendment 1 (15th April 2015) 2. Amendment 2 (Number: 2015/991-32. 10th June 2015) 3. Amendment 3 (Number: 2015/2056-32. 30th November 2015) 4. Amendment 4 (Number: 2016/1191-32. 14th June 2016) 5. Amendment 5 (Number: 2016/2531-32. 4th January 2017) 6. Amendment 6 (Number: 2017/638-32. 28th Mars 2017) 7. Amendment 7 (Number: 2018/1012. 30th May 2018) |
| **22. Completion date** | Twelve months follow-up for the last patient included is due to July 2020. Long-term follow-up via central registry in Sweden (survival and health economic data) for at least three years. |
| **23. Summary Results** | Not available yet. |
| **24. IPD sharing statement** | The datasets are available from the Chief Investigator on reasonable request. We plan to do an of individual patient data meta-analysis for EFFECTS, FOCUS and AFFINITY. In addition, we plan to update the Cochrane systematic review of selective serotonin reuptake inhibitors for stroke recovery. |
