# Supplementary material for: Update on the EFFECTS study of fluoxetine for stroke recovery: a randomised controlled trial in Sweden
Source: Trials. 2020 Feb 28;21:233. doi: 10.1186/s13063-020-4124-7 (PMC7048055; doi:10.1186/s13063-020-4124-7)
Supplement: Supplementary file 3 — Additional file 3. Research Ethical Committee and the Swedish Medical Product Agency approvals for EFFECTS. [file 13063_2020_4124_MOESM3_ESM.pdf]

**Appendix**

DECISION

Ref. 2013/1265-31/2

EPN STOCKHOLM

Applicant: Danderyd Hospital
Authorised representative: Karin Malmqvist
Project: EFFECTS – Establishing the effect and safety of fluoxetine in the case of stroke – a randomised placebo-controlled study of 1,500 patients.
Researcher who will carry out the project: Veronica Murray

At the meeting of 21 August 2013, the board decided that the applicant should add to the case in accordance with the following:

The board requests that the applicant adds to the case in accordance with the following:

1. "Ethics Committee" should be changed to the Ethical Review Board.

2. There should be a safety monitoring board to evaluate any side-effects during the course of the study. Is there such a board?

3. The participant information should begin with information about the fact that the participant is included in a study.

The board handed over to the scientific secretary to decide on the case once the addition has been made.

Since the applicant has submitted the requested additions, the scientific secretary decides as follows.

## DECISION

The board approves the research.

On behalf of the board
[signed] 30 September 2013
Anders Björkman
Scientific secretary

Translated by
Språkservice
Reference: NKZ70
Malmö, Sweden: 20170920

KHOLM
**Street address** Nobels väg 9 Solna
**Telephone** +46 (0)8 524 864 00 (switchboard)
**Fax** +46 (0)8 524 86699
**E-mail** kansli@stockholm.epn.se
**Website** www.epn.se
Website www.epn.se

**Karolinska Institutet**

**29 March 2015**



**Department of Clinical Sciences**
**Danderyd Hospital**
Surgery Unit
Professor Erik Näslund
Head of Department, consultant

Regional Ethical Review Board in Stockholm
Karolinska Institutet
171 77 Stockholm

Regionala [illegible]vnings-
nämnden i Stockholm
Inkom: 2015 -03- 29
Dnr:

## Addition 2013/1265-31/2

The undersigned wishes to add to the above application in relation to the EFFECTS study.

1. Main responsibility for the study was held by Veronica Murray at the Department of Clinical Sciences, Danderyd Hospital. Tragically, Veronica Murray died on 27 December 2014. The undersigned Head of Department has appointed Dr Erik Lundström from Karolinska Solna as the person with main responsibility for the project. A CV and a list of publications for Dr Erik Lundström are attached.
2. The latest version of the study protocol is also attached, in which a few names from the steering group have been changed in view of Veronica Murray's death, and the health economics sub-study has been clarified.
3. Resource certification from Karolinska Huddinge, St Göran's Hospital, Danderyd Hospital, Karolinska Solna, Hässleholm, Uppsala University Hospital, Skaraborg Hospital. Additional hospitals will be added.

15 April 2015

Regards
[signed]
Erik Näslund
Head of Department

**APPROVED**
**[signed]**
**Pär Sparén**
**Scientific secretary**
**Regional Ethical Review Board in Stockholm**

A fee of SEK 2,000 has been paid via Karolinska Institutet.

**Postal address**
Surgery Unit
Department of Clinical Sciences, Danderyd Hospital
Karolinska Institutet
182 58 Stockholm

Co. reg. no. 202100 2973

**Street address**
Danderyd Hospital
Stockholm

**Telephone**
+46 (0)8 12355000, switchboard
+46 (0)8 12355017, direct
**Fax**
+46 (0)8 12357786

**E-mail**
Erlk.Naslund@ki.se
**Website**
ki.se

Translated by
Språkservice
Reference: NKZ70
Malmö, Sweden: 20170920

**Department of Clinical Neuroscience**
**Karolinska University Hospital, Solna**
**Neurology, R3:04**
**Erik Lundström**
**Doctor of Medicine, Section Consultant**

Regional Ethical Review Board in Stockholm
Karolinska Institutet
171 77 Stockholm

Regional Ethical Review Board in Stockholm
Received: 4 June 2015

Ref.: 2015/991-32

## Addition to EFFECTS, reference number 2013/1265-31/2

The undersigned wishes to add to the above application in relation to the EFFECTS study.

We have now carried out the feasibility phase of EFFECTS, and according to the earlier research plan we then planned to evaluate the protocol and make the necessary changes according to the experience obtained.

In order to facilitate the Ethical Review Board's assessment, I have attached both the current version of the protocol (version 4.5, date 15 March 2015) and the patient consent document (version 4.3)

Page numbers refer to version 4.5 of the protocol. New text is marked in red. This text colour will be reverted if the Ethical Review Board approves these additions. The following changes have been made between versions 4.5 and 4.6 of the protocol:

a) Change to patient consent. That the patient consents to us obtaining information from registers on care consumption, and clarification of the side effects.
Reason: This provides more secure data about health economics, while at the same time making things simpler for the study and reducing the burden of questions to patients and relatives.

b) Page 19. Paragraph 1, changed from "more than 7 000 observed" to "up to 6 100 observed patients"
Reason: The pooled number of individuals in EFFECTS, FOCUS and AFFINITY will be a maximum of 6,100 patients.

c) Page 21 paragraph 2.2.2. Addition of using register data. The sentence "Long-term data will also be retrieved from the Cause of Death Register and the National Patient Register, up to 3 years after inclusion of the last patient."
Reason: This provides more secure data about health economics, while at the same time making things simpler for the study and reducing the burden of questions to patients and relatives.

d) Page 23, paragraph 1, removal of the sentence "a printed eCRF, and a copy of all forms used. All forms will be possible to download from the trial website."
Reason: It will be possible to print out our eCRF from the website, and we do not therefore deem it necessary to have it in the Investigator Site File.

e) Pages 30-31. The sentence "The total amount of capsules for six months is 186 capsules of fluoxetine 20mg and 186 capsules of matching placebo" is removed. Change number of capsules to 100 (from 107 and 93).

**Postal address/Street address**
**Street address**
Karolinska University Hospital, Solna
Neurology R3:04
171 76 Stockholm
Co. reg. no. 202100 2973

**Telephone**
+46 (0)8 517 70 000, switchboard
+46 (0)8 517 746 97, direct
**Fax**
+46 (0)8 517 737 57

**E-mail**
erik.lundstrom@ki.se
**Website**
ki.se
www.effects.se

Removal of “93 capsules + 14 in back-up, total”, change to “100 capsules”
Change from "(93 capsules)” to “(100 capsules)”
Point 9.8.1, remove “in the patient diary”
Reason: Correction to the right number of capsules. Patients do not have any diaries for side effects.

f) Page 35. Adjustment and correction of errors in Table 10.1. STUDY ASSESSMENT SCHEDULE. Addition of time intervals for the various follow-ups.

g) Page 36, 10.2, last sentence of paragraph 1 “The patient and relatives will receive a diary in which they are encouraged to record the date and nature of any adverse events.” is removed.
Reason: Patients do not have any diaries for side effects.

h) Page 36. Under the heading Alert of Adverse Reactions, the following sections are removed: “… will be sent or faxed to the coordinating center…” and “… If no discharge form is received by 6 weeks the center will be prompted by fax or email to send the discharge form. If the patient is still in hospital the local research team will be asked…”

Regarding the system for event reporting, the sentence: “At these follow ups the GP or other responsible physician will be asked by the local EFFECTS team about adverse events.” is removed.
Reason: We want to simplify the process for the local centre. In order to maintain security, we will encourage patients and relatives to call the local centre to report. Our experience during the pilot phase is that this system works better – both patients and relatives find it easier to contact their local physician or nurse.
The reference saying that we will have a special system with pre-stamped envelopes and a web-based solution for patients and relatives is thus omitted.

Under point 10.3, we have re-worded the text so that it corresponds with the follow-up carried out (typographical error in the protocol on this page). We are therefore adjusting the text to: face-to-face follow-up locally at 6 months and additional central follow-up (survey) at 6 and 12 months.
We will not have any web-based follow-up available for patients and relatives.

i) Page 37. Sample size calculation: Minor modification, since the sister study AFFINITY is expected to include 1,600 patients (not 1,500), the total included in the study is adjusted to 6,100 (not 6,000).

The following text is removed:
“The trial steering committee (TSC) will review the target sample size at the end of the feasibility phase and adjust this based on:

- Advice from the DMC
- Accruing data on
    - o the enrolment into specific pre-specified subgroups
    - o completeness of follow up
    - o distribution of mRS categories in the population of enrolled subjects (i.e. both treatment groups combined), overall and in specific patient categories (e.g. those with motor deficits, aphasia, etc.)

For example, if the distribution of mRS is different to that anticipated, then the sample size might need to be increased. This approach has the advantage that such sample size adjustments can be made without reference to the accumulating blinded data, and avoids the need for conditional power calculations which can be unreliable.”
Reason: This text is not correct.

j) Page 39. Motor function – NIHSS, speech – NGTA
The following sentence is removed: “In this case the total population will be 1550; if however trial eligibility has had to be changed we will report the 1500 from the main phase as main findings, and the 50 from the feasibility phase separately.”
Reason: We do not use the Fugl-Meyer Scale or ANELT (typographical error in the protocol on this page).

k) Page 40. Adjustment of the number of EQ5D-5L measurements during the main phase: a reduction from having measured EQ5D-5L during the pilot phase on inclusion on 6 occasions (1 week, 4 weeks, 3 months, 6 months and 12 months) to measuring at 4 measurement points (inclusion, and 3, 6 and 12 months).
Reason for this adjustment: We do not need 6 measurement points for quality of life, and we want to reduce the burden for patients.

l) Page 43, Section 15.3.1, paragraph 3. We are adjusting the wording to make it clearer that SUSAR must be reported via the helpline within 24 hours instead of by fax. The sentence now reads “SUSAR should be reported to the helpline (073-663 74 44) within 24 h”.
The sentence “and must sign the EFFECTS trial prescription form for the trial medication.” is deleted. Does not apply.

m) Page 52. Clarification that only the most recent version of the research protocol needs to be included in the Investigator Site File. The following sentence is added: “Every center must have the latest version of the protocol in their Investigator Site File.”

Changes to the form:
m) Remove identity for MoCA. Reason: Not compatible with GCP.

n) Print-out form: Remove “If there have been changes to the medication at baseline”. Medications must instead be listed when printing out the form.
Reason: The previous reasoning was a little unclear. We are making this change to make it clearer and simpler.

o) Changes to “Patient and relative information 18 May 2015 version 3” – clarification of possible side effects of fluoxetine, and request to be able to use register data. Changes to the text are marked in red in the accompanying document. This red marking will then be removed. The text reads:

“I also give my consent for information about being signed off sick, care-related consumption of resources and survival to be obtained from public registers. All data will be processed in anonymised form.
Your personal data will be dealt with in accordance with the Swedish Data Protection Act. Danderyd Hospital is responsible for your personal data. You are entitled to receive an extract of your personal data once a year, and can contact Eva Isaksson (tel. no. +46 (0)8 123 576 93) to obtain this.”

Translated by
Språkservice
Reference: NKZ70
Malmö, Sweden: 20170920

Appendices

| **Current version** | **Updated version** |
| --- | --- |
| Study Protocol<br>Version 4.5, date 15 March 2015 | Study Protocol<br>Version 4.6, date 18 May 2015 |
| Patient and relative information version 4.2<br>25 April 2013 version 2 | Patient and relative information<br>18 May 2015 version 3 |

Resource letters from the following hospitals:
Mora General Hospital, Falu General Hospital
Lidköping[1)]/Norrtälje, Kristianstad

Stockholm, date as above

Erik Lundström
Chief Investigator EFFECTS

A fee of SEK 2,000 has been paid via Karolinska Institutet.

Resource letters have previously been submitted for: Danderyd Hospital, Karolinska Hospital (Solna), Hässleholm, Skaraborg Hospital Skövde, Uppsala University Hospital, Karolinska Hospital (Huddinge) and Capio St Göran's Hospital

1) Lidköping refers to “Skaraborg Hospital, Lidköping”

Translated by
Språkservice
Reference: NKZ70
Malmö, Sweden: 20170920

APPROVED 10 June 2015
[signed]
Pär Sparén
Scientific secretary
Regional Ethical Review Board in Stockholm

Translated by
Språkservice
Reference: NKZ70
Malmö, Sweden: 20170920

Karolinska Institutet

**Department of Clinical Neuroscience**

Karolinska University Hospital, Solna
Neurology, R3:04 Erik Lundström
Doctor of Medicine, Section Consultant

Regional Ethical Review Board in Stockholm
Karolinska Institutet
171 77 Stockholm

**Amendment 3 for the EFFECTS study.**

Regionala etikprövningsnämnden i Stockholm
Inkom: 2015 -11- 25
Dnr:

**2015/2056-32**

**Re: EFFECTS ESTABLISHING THE EFFECTS(S) AND SAFETY OF FLUOXETINE INITIATED IN THE ACUTE PHASE OF STROKE**

**Main application: Ref.: 2013/1265-31/2. Date 30 September 2013**
**Amendment 1: Date: 15 April 2015**
**Amendment 2: Ref.: 2015/991-32. Date 10 June 2015**

**A:** As an addition to the previously approved application, the following centres will include patients in the study (resource letters attached):

1. Rehab Station Stockholm
2. Mälar Hospital Eskilstuna
3. Halland Hospital Halmstad
4. Skåne University Hospital Malmö
5. Helsingborg General Hospital
6. Norrland University Hospital Umeå
7. Visby General Hospital
8. Sundsvall Hospital.

APPROVED 30 November 2015
[signed]
Pär Sparén
Scientific secretary
Regional Ethical Review Board in Stockholm

We have previously submitted resource letters for: Danderyd Hospital, Karolinska Hospital (Solna), Hässleholm, Skaraborg Hospital Skövde, Uppsala University Hospital, Karolinska Hospital (Huddinge) and Capio St Göran's Hospital, Mora General Hospital, Falu General Hospital, Lidköping, Norrtälje and Kristianstad.

**Clarification in the Research Protocol; updated to version 4.7.** After having carried out the pilot phase, we have made certain adjustments to the Research Plan.

Translated by
Språkservice
Reference: NKZ70
Malmö, Sweden: 20170920

B: When it comes to quality of life, the following is stated in our protocol (page 39 in Research Protocol version 4.6)

> Self-reported quality of life will during the pilot phase, measured at baseline, 1 week, patient or proxy), 4 weeks, 3 moths [sic], 6 months, and at 12 months of follow up will be measured using the EuroQoL 5 Dimensions (EQ5D-5L) scale.
>
> In the main phase, EQ5D will be measured at inclusion, at 6 and 12 months follow-up.

After having received a number of questions from participating centres and our monitors, we would like to clarify the sentence about the main phase. First, a little background. We have close cooperation with our sister study FOCUS in Edinburgh. FOCUS measures EQ5D at 6 and 12 months centrally via a survey that is sent to the patient's home. In this follow-up, only the question section of EQ5D is used, not the VAS thermometer (page 2 in EQ5D). The reason for this is that an additional survey – the Stroke Impact Scale (SIS) – includes a VAS thermometer. We have been concerned that the patients would conflate the different thermometers. At the same time, we have been keen to be able to pool data with FOCUS (Edinburgh). This means that in the two central forms, our data and Edinburgh's data are identical, the questions in EQ5D.

At the same time, our ambition has been to make the health economic analysis in Sweden clearer. We have therefore introduced EQ5D <u>on inclusion and at the local repeat visit, at 6 months</u>. Because we wanted it to be possible to compare inclusion with the 6-month check, we used the entire EQ5D instrument, i.e. the 5 questions including the VAS thermometer at:

a) Local measurement of the entire EQ5D on inclusion (not included in Edinburgh)
b) Local measurement of the entire EQ5D at 6 months (not included in Edinburgh)

In order for this to be completely clear, we have made certain changes in 10.1 on page 35 of the Research Protocol. The text marked in red and the figures are stated to highlight what is commented on under the table. We will change the time intervals to months (after 1 week – see the heading row marked in red). Instead of writing 4 weeks, we are now writing 1 month, etc. The figures in the table and the text colour will be removed in the published protocol.

Translated by
Språkservice
Reference: NKZ70
Malmö, Sweden: 20170920

Karolinska Institutet

Regional Ethical Review Board in Stockholm

Received 9 June 2016

Ref.: 2016/1191-32

**Department of Clinical Neuroscience**

Karolinska University Hospital, Solna Neurology, R3:04 Erik Lundström
Doctor of Medicine, Section Consultant

Regional Ethical Review Board in Stockholm
Karolinska Institutet
171 77 Stockholm

**Amendment 4 for the EFFECTS study.**

**Re.: EFFECTS ESTABLISHING THE EFFECTS(S) AND SAFETY OF FLUOXETINE INITIATED IN THE ACUTE PHASE OF STROKE**

**Main application: Ref.: 2013/1265-31/2. Date 30 September 2013**
**Amendment 1: Date: 15 April 2015**
**Amendment 2: Ref.: 2015/991-32. Date 10 June 2015**
**Amendment 3: Ref.: 2015/2056-32. Date 30 November 2015**

A: As an addition to the previously approved application, resource letters are submitted for:

1. Sahlgrenska Hospital, Gothenburg
2. Högsbo Hospital, Gothenburg
3. Stora Sköndal
4. Östersund Hospital
5. Alingsås Hospital
6. Ängelholm Hospital
7. Stockholm Nursing Home
8. Skåne University Hospital, Lund
9. Örebro University Hospital

We intend to submit resource letters, provided that we have carried out the process described in accordance with point B below, i.e. provided that they meet the requirements for participation in EFFECTS, for Norra Älvsborg County Hospital, Östra Hospital, Sunderby Hospital, Skellefteå General Hospital, Karlstad Central Hospital, Södertälje Hospital, Västmanland Hospital Västerås, Kullbergska Hospital, Ryhov County Hospital Jönköping, Blekinge Hospital Karlshamn, Blekinge Hospital Karlskrona, Kalmar County Hospital, Halland Hospital, Varberg, Södra Älvsborg Hospital, Bromma Geriatric Clinic and

Translated by
Språkservice
Reference: NKZ70
Malmö, Sweden: 20170920

Kungshomen Geriatric Clinic.

Translated by
Språkservice
Reference: NKZ70
Malmö, Sweden: 20170920

**Regional Ethical Review Board in Stockholm**

**Received: 21 December 2016**

**Ref. no.: 2016/2531-32**

**Karolinska Institutet**

**Department of Clinical Neuroscience**

Karolinska University Hospital, Solna Neurology,
R3:04
Erik Lundström
Doctor of Medicine, Section Consultant
erik.lundstrom@ki.se
Tel. +46 (0)707 677 411, +46 (0)8 517 746 97

Regional Ethical Review Board in Stockholm
FE 289
171 77 STOCKHOLM
E-mail: kansli@stockholm.epn.se
Telephone: +46 (0)8 524 870 00

Stockholm, 21 December 2015

**Amendment 5 for the EFFECTS study, and**
**Annual safety report for EFFECTS EFFECTS: ESTABLISHING THE EFFECTS(S) AND SAFETY OF FLUOXETINE INITIATED IN THE ACUTE PHASE OF STROKE. Relates to the period 20 October 2014 to 31 October 2016**

**EudraCT no.: 2011-006130-16**
**EPN no.: Ref. no.: 2013/1265-31/2. Date 30 September 2013**
**Amendment 1: Date: 15 April 2015**
**Amendment 2: Ref. no.: 2015/991-32. Date 10 June 2015**
**Amendment 3: Ref. no.: 2015/20156-32. Date 30 November 2015**
**Amendment 4: Ref. no.: 2016/1191-32. Date 14 June 2016**

This is amendment 5 for EFFECTS. It includes year 2 of the annual safety report. This is a copy of the safety report that is sent to the Swedish Medical Products Agency. It also includes some amendments, **points A-C below**. The report covers the period since the study started (20 October 2014). In addition to the Swedish Medical Products Agency and the Ethical Review Board, the safety report has also been sent to the steering group and the Safety Committee for EFFECTS, the heads of the Department of Clinical Sciences at Danderyd Hospital and the Department of Clinical Neuroscience (CNS) at Karolinska Institutet, Professor Erik Näslund and Jan Hillert, and our monitors at Karolinska Trial Alliance.

During the year, the Safety Committee has held two meetings and has notified the Chief Investigator that EFFECTS can continue as planned since the study meets the necessary safety requirements.

**A:**

As an addition to the previously approved application, resource letters are submitted for:

1. Norra Älvsborg County Hospital Trollhättan, Bromma Geriatric Clinic and Västmanland Hospital Västerås

Additional centres that may be included during 2017 are: Lindesberg (will be included), Dalen Hospital, Sollefteå Hospital, Enköping, Kalmar, Eksjö, Värnamo, Östra Hospital, Borås, Sunderby Hospital, Skellefteå, Karlstad, Södertälje, Kullbergska, Jönköping, Karlskrona, Karlshamn and Varberg.

Translated by
Språkservice
Reference: NKZ70
Malmö, Sweden: 20170920

We have previously submitted resource letters for: Danderyd Hospital, Karolinska Hospital (Solna), Hässleholm, Skaraborg Hospital Skövde, Uppsala University Hospital, Karolinska Hospital (Huddinge), Capio St Göran's Hospital, Mora General Hospital, Falu General Hospital, Lidköping, Norrtälje, Kristianstad, Rehab Station Stockholm, Mälar Hospital Eskilstuna, Halland Hospital Halmstad, Skåne University Hospital Malmö, Helsingborg General Hospital, Skåne University Hospital, Lund, Norrland University Hospital Umeå, Visby General Hospital, Sundsvall Hospital, Sahlgrenska Hospital, Gothenburg, Högsbo Hospital, Gothenburg, Stora Sköndal, Östersund Hospital, Alingsås Hospital, Ängelholm Hospital, Stockholm Nursing Home and Örebro University Hospital Rehabilitation Medicine Clinic.

**B:**

**Major amendments, updates in the Research Protocol to version 4.8**

1. The company that manufactures fluoxetine has updated its SPC. They now state that if metoprolol is used in the case of heart failure, fluoxetine is contraindicated. EFFECTS' Steering Committee and Safety Committee have made the assessment that this applies to serious heart failure, that it may be clinically significant in the case of more advanced heart failure (NYHA Grad IIB – IV) and especially in higher doses, and that in the case of simultaneous treatment with metoprolol and fluoxetine one should be attentive to the interaction and should follow up on the patient soon after inclusion with clinical monitoring including ECG. In the annual safety report to the Swedish Medical Products Agency, we have carried out a thorough analysis in relation to this problem, and it does not recur here. In summary, EFFECTS' steering group is of the opinion that this did not give cause for any change to the study, as this falls under the exclusion criterion *pharmaceuticals that have significant interactions with SSRIs*. All centres have been notified of this serious interaction, and we have clarified this exclusion criterion through the following addition to our research plan.

   "Fluoxetine is contra-indicated in combination with metoprolol used in cardiac failure New York Heart Association Grade IIIB and IV. At higher doses of metoprolol used in heart failure indication one should be vigilant of the interaction and early after enrollment monitor the patient with clinical monitoring including ECG."

   Page 25 in the Research Plan

2. We have previously written that participation in another CTIMP does not automatically rule out participation in EFFECTS, but that it is important not to overburden patients with studies. In the section about co-enrolment, we now mention the TIMING study and write:

   "It is allowed to co-enroll patients in EFFECTS and the TIMING study. The intervention in TIMING is early vs delayed start of NOAC in patients with acute stroke and atrial fibrillation. Thus, all patients would receive NOAC either <=4 days or > 5 days from the acute stroke."

   Page 26 in the Research Plan

3. We have noted that our protocol has not specified how long we recommend stopping in the event of suspected side effects and whether we will permit restarting medication after a longer stop. We have now clarified this in the updated version. We write:

Translated by
Språkservice
Reference: NKZ70
Malmö, Sweden: 20170920

"We recommend coming off IMP for 14 days to see if the symptoms resolve. If they do then ideally they would restart to see if symptoms return. However, we recognize very few patients are prepared to do so. All stops (temporary and permanent) of the IMP must be registered in the e-CRF. There is not any limit for how long a temporary stop might be."

Page 25 in the Research Protocol.

**C:**

**Minor amendments, updates to version 4.8**

**Page 1**
Added reference number and EFFECTS study number in the Clinicaltrials.gov database to Amendments 3 and 4. Changed the protocol version to version 4.8 and the date to 21 December 2016.

**Page 52**
In the protocol, we clarify that the amendment of a centre in the study does not need to be sent out to all centres as a protocol amendment. This is communicated in connection with major protocol changes and electronically via the weekly newsletter and on the study's website (www.effects.sehttp://www.effects.se/). We write:

"Amendment relating to the addition of centers in the study do not need to be sent out to all centers as a protocol amendment. This is communicated in connection with major protocol changes and electronically via the newsletter and on the study website (www.effects.se)."

A fee of SEK 2,000 will be paid this week, stating the reference:
Amendment 5 EFFECTS/Lundström

[signed]
Erik Lundström
Chief Investigator EFFECTS

APPROVED 4 January 2017

[signed]
Pär Sparén
Scientific secretary
Regional Ethical Review Board in Stockholm

Appendices:

Copies of Resource certification for new centres
Arlig_sakerhetsrapport_EFFECTS_2016 including 2 appendices marked with *
* Appendix 1 EFFECTS 2016
* Appendix 2 EFFECTS 2016
EFFECTS Protocol version 4 8 EU no. 2011-006130-16

Translated by
Språkservice
Reference: NKZ70
Malmö, Sweden: 20170920

Regional Ethical Review Board in Stockholm
Received: 28 March 2017
Ref. no.: 2017/638-32

Karolinska Institutet

**Department of Clinical Neuroscience**

Karolinska University Hospital, Solna Neurology, R3:04
Erik Lundström
Docent, Section Consultant
erik.lundstrom@ki.se
Tel. +46 (0)707 677 411, +46 (0)8 517 746 97

Regional Ethical Review Board in Stockholm
FE 289
171 77 STOCKHOLM
E-mail: kansli@stockholm.epn.se
Telephone: +46 (0)8 524 870 00

Stockholm, 24 March 2017

**Amendment 6 for the EFFECTS study**

Approved 28 March 2017
[Signed]
Scientific secretary
Regional Ethical Review Board in Stockholm

**EudraCT no.: 2011-006130-16**
**EPN no.: Ref. no.: 2013/1265-31/2. Date 30 September 2013**
**Amendment 1: Date: 15 April 2015**
**Amendment 2: Ref. no.: 2015/991-32. Date 10 June 2015**
**Amendment 3: Ref. no.: 2015/20156-32. Date 30 November 2015**
**Amendment 4: Ref. no.: 2016/1191-32. Date 14 June 2016**
**Amendment 5: Ref. no.: 2016/2531-32). Date 4 January 2017**

As an addition to the previously approved application, resource letters are submitted for:

1. Dalen Hospital and Lindesberg General Hospital. For reference, we have changed the PI at Skövde from Erik Bertholds to Björn Cederin, and at Karolinska Hospital Huddinge from Ioanna Markaki to Maria Lantz. This has been updated in the delegation lists.

Additional centres that may be included during 2017 are: Hudiksvall, Kalmar, Eksjö, Värnamo, Östra Hospital, Borås, Sunderby Hospital, Skellefteå, Karlstad, Södertälje, Kullbergska, Jönköping, Karlskrona, Karlshamn and Varberg.

We have previously submitted resource letters for: Danderyd Hospital, Karolinska Hospital (Solna), Hässleholm, Skaraborg Hospital Skövde, Uppsala University Hospital, Karolinska Hospital (Huddinge), Capio St Göran’s Hospital, Mora General Hospital, Falu General Hospital, Lidköping, Norrtälje, Kristianstad, Rehab Station Stockholm, Mälar Hospital Eskilstuna, Halland Hospital Halmstad, Skåne University Hospital Malmö, Helsingborg General Hospital, Skåne University Hospital Lund, Norrland University Hospital Umeå, Visby General Hospital, Sundsvall Hospital, Sahlgrenska Hospital, Gothenburg, Högsbo Hospital, Gothenburg, Stora Sköndal, Östersund Hospital, Alingsås Hospital, Ängelholm Hospital, Stockholm Nursing Home, Örebro University Hospital Rehabilitation Medicine Clinic, Norra Älvsborg

Translated by
Språkservice
Reference: NKZ70
Malmö, Sweden: 20170920

Translated by
Språkservice
Reference: NKZ70
Malmö, Sweden: 20170920

**Major amendments, updates in the Research Protocol to version 4.9**

1. Our primary outcome measure is an ordinal scale called the modified Rankin Scale (mRS). The scale, which goes from 0 (no symptoms) to 6 (dead), is the most common outcome measure for stroke. The mRS is most commonly carried out at a repeat visit, but it can also be done by telephone or via a survey. Carrying out surveys during repeat visits can be time-consuming, particularly in the case of large studies, and our colleagues in Edinburgh have therefore developed a scale called the *simple modified Rankin Scale questionnaire* (smRSq). This consists of five questions, and can be carried out as a survey or by telephone. smRSq is validated in English, but not in Swedish. In our research plan, we stated that we planned to carry this out during 2013 – see below. However, because we have been forced to focus on other issues (preparing randomisation systems, eCRF, inclusion of patients in the study), we have not been able to carry out the planned study. Since several years have passed since we applied, we believe that it is important to clarify our position on this matter to the Ethical Review Board.

We wrote the following in version 4.8 of the research plan, on page 22. The wording remains unchanged since the first application, which was approved on 30 September 2013:

"Modified Rankin Scale (mRS) (based ordinal analysis to maximize power and to avoid problems including patients with an mRS > 2 prior to their stroke) at 6 months after randomization. Patient who die would be attributed a score of 6 for this analysis.

The mRS is an extremely simple, time efficient measure with well-studied reliability used to categorize level of functional outcome. It has been used extensively in large, multicentre stroke trials.

Any misclassification of patients into an inappropriate mRS category may reduce the power of the trial. To minimize misclassification and intermodality differences we will use the simple modified Rankin Scale questionnaire (smRSq) described by Bruno and colleagues. This has been delivered by both telephone and postal questionnaires and has been completed by patients and proxies (Bruno 2010, 2011) (Dennis 2012) (Lundström in early manuscript 2013)."

What we now intend to do is to investigate whether the survey that we sent out at 6 and 12 months gives similar results compared with a traditional assessment during a repeat visit.

This does not involve any additional burden for the patient compared with how we do things now. Every participant in the study already answers the five questions that form the basis for smRSq. What is being added is a number of physicians and nurses carrying out a traditional assessment of mRS at the 6 month repeat visit.

All the information required in order to carry out a regular mRS is obtained during the ordinary repeat visit. I have personally tried out doing this at a number of repeat visits, and it does not make the repeat visit any longer or more difficult for the patient.

Translated by
Språkservice
Reference: NKZ70
Malmö, Sweden: 20170920

However, since the planned comparison between smRS and mRS has not yet been carried out, we would like to apply with this amendment to carry out the sub-study.

This will affect a total of 65 individuals.

The method for carrying out a study within a study is called *Study Within A Trial* (SWAT) in English (Anon 2012). We intend to register this study in a register called the Northern Ireland Hub for Trials Methodology.

The changes in version 4.9 of the research plan are marked in red below:

"Modified Rankin Scale (mRS) (van Swieten 1988) (based ordinal analysis to maximize power and to avoid problems including patients with an mRS > 2 prior to their stroke) at 6 months after randomization. Patient who die would be attributed a score of 6 for this analysis.

The mRS is an [sic] simple, time efficient measure with well-studied reliability used to categorize level of functional outcome. It has been used extensively in large, multicentre stroke trials.

Any misclassification of patients into an inappropriate mRS category may reduce the power of the trial. To minimize misclassification and intermodality differences we will use the simple modified Rankin Scale questionnaire (smRSq) described by Bruno and colleagues. This has been delivered by both telephone and postal questionnaires and has been completed by patients and proxies (Bruno 2010, 2011; Dennis 2012). The smRSq has been validated in English (Bruno 2010, 2011; Dennis 2012) but not in Swedish. We are planning to test the agreement of the Swedish *small modified Rankin Scale questionnaire with face-to-face modified Rankin Scale.* (Lundström manuscript synopsis 2017).

Synopsis of manuscript with preliminary title: *Agreement of the Swedish small modified Rankin Scale questionnaire with face-to-face modified Rankin Scale*

The smRSq is sends [sic] to patients by the Trial Manager Assistant (TMA) at 6 and 12 month post randomisation. If the patient does not answer, the TMA contacts the patient by phone and reminds them to send in the questionnaire. If they have difficulty answering for themselves TMA helps them fill in the form by phone.

*Statistics*

*Number of patients*

The primary aim of the study is to evaluate whether the mRs-score measured by the smRSq differs from a mRS-score measured by a clinician. It has been defined that one step or more disparity in the mRs-score is a significant difference. A study of similar character has never been performed before and due to the nature of the study, an initial study, the sample size is not formulated in the guise of power, risk level, or clinical difference. The number of patients participating in the study is therefore primarily chosen for clinical reasons, not statistical, and 60 patients will be included in the study. In order to compensate for included patients not valid for efficacy analysis it is planned to enrol up to 65 patients in the study in order to have 60 patients valid for efficacy analysis. The attrition rate is estimated to be about 6%.

Translated by
Språkservice
Reference: NKZ70
Malmö, Sweden: 20170920

*Statistical methods and data management*

Statistical comparisons in order to test differences between dependent observations will be made by use of pair-wise Student's t-test for correlated means and statistical comparisons between two independent groups will be made by use of the Student's t-test for uncorrelated means., [sic] after validation for normal distribution by use of the Shapiro Wilk test. The Pearson correlation coefficient will be used in order to test independence between variables. In addition to that descriptive statistics will be used to characterize the data. All analyses will be carried out by use of the SAS system (The SAS system for Windows 9.4., SAS Institute Inc, Cary, NC, USA.) and the 5% levels of significance will be considered. In the case of a statistically significant result the probability value (p-value) will be given. The results will be presented in a cross table. The proportion of full agreement will be given in percent and 95% Confidence Interval, as well as weighted and not weighted Kappa value.

A fee of SEK 2,000 will be paid, stating the reference:
Amendment 6 EFFECTS/Lundström

Erik Lundström
Chief Investigator EFFECTS

Appendices:
Copies of Resource certification for new centres
EFFECTS Protocol version 4 9 EU no. 2011-006130-16

References

**Anon. Education section - Studies Within A Trial (SWAT). Journal of Evidence-Based Medicine 2012; 5(1): 44-5**

**Bruno A, Shah N, Lin C, Close B, Hess D, Davis K, Baute V, Switzer J, Waller J, Nichols F. Improving modified Rankin scale assessment with a simplified questionnaire. Stroke. 2010;41:1048-1050**

**Bruno A, Akinwuntan AE, Lin C, Close B, Davis K, Baute V, Aryal T, Brooks D, Hess D, Switzer J, Nichols F. Simplified Modified Rankin Scale Questionnaire: Reproducibility Over the Telephone and Validation With Quality of Life. Stroke. 2011;42:2276-2279.**

**Dennis M, Mead G, Doubal F, Graham C. Determining the modified Rankin score after stroke by postal and telephone questionnaires. Stroke 2012; 3:851-3**

**van Swieten JC, Koudstaal PJ, Visser MC, Schouten HJ, van Gijn J. Interobserver agreement for the assessment of handicap in stroke patients. Stroke. 1988 May;19(5):604-7**

Translated by
Språkservice
Reference: NKZ70
Malmö, Sweden: 20170920

**Department of Clinical Neuroscience**

Karolinska University Hospital, Solna Department of Neurology, R3:04
Erik Lundström
Associate Professor,
Senior Consultant
erik.lundstrom@ki.se
Tel. +46 (0)707-677 411

Regional Ethical Review Board in Stockholm
FE 289
171 77 STOCKHOLM
E-mail: kansli@stockholm.epn.se
Telephone: +46 (0)8-524 870 00

Stockholm, 10 May 2018

**Amendment 7 for the EFFECTS study**

**EudraCT no: 2011-006130-16**
**EPN no: Ref. no: 2013/1265-31/2. Date 30/09/2013**
**Amendment 1: Date: 15/04/2015**
**Amendment 2: Ref. no: 2015/991-32. Date 10/06/2015**
**Amendment 3: Ref. no: 2015/20156-32. Date 30/11/2015**
**Amendment 4: Ref. no: 2016/1191-32. Date 14/06/2016**
**Amendment 5: Ref. no: 2016/2531-32. Date 04/01/2017**
**Amendment 6: Ref. no. 2017/638-32. Date 28/03/2017**

As a supplement to the already approved application, a resource letter is submitted for Hudiksvall Hospital.

For information purposes, we have switched PI in the following places: Rehab Station Stockholm (from Sabahudin Bjelak to Liisa Hopia), Stora Sköndal (from Mehran Taklif to Anna Sjöström), Norrtälje Hospital (Ann Engquist to Moa Gunnarsson). This has been updated in the delegation lists. We have closed the following centres: Visby, Högsbo, Lidköping Örebro and Bromma Geriatric Hospital. We will also change PI at Karolinska Hospital, Solna in the spring as I will be ending my employment there. I am currently on leave from my position as a senior consultant at Karolinska University Hospital in order to try out a corresponding role at Uppsala University Hospital. However, I am still affiliated with KI, just as before, but specialist doctor Bjarni Gudmundsson will assume responsibility as PI. Furthermore, we have not changed anything regarding sponsorship or finances – this remains at KI. All this is supported and approved by the EFFECTS steering committee and responsible head of department at KI, Professor Erik Näslund.

Previously, we submitted resource letters for: Danderyd Hospital, Karolinska Hospital (Solna), Hässleholm, Skaraborg Hospital Skövde, Uppsala University Hospital, Karolinska Hospital (Huddinge), Capio S:t Görans Hospital, Mora Hospital, Falu Hospital, Lidköping, Norrtälje, Kristianstad, Rehab Station Stockholm, Mälarsjukhuset Hospital Eskilstuna, Hallands Hospital Halmstad, Skåne University Hospital

Malmö, Helsingborg Hospital, Skåne University Hospital Lund, Norrland's University Hospital Umeå, Visby Hospital, Sundsvall Hospital, Sahlgrenska University Hospital Gothenburg, Högsbo Hospital Gothenburg, Stora Sköndal, Östersund Hospital, Alingsås Hospital, Ängelholm Hospital, Stockholm Sjukhem, Örebro University Hospital Rehab Medicine, Northern Älvsborg County Hospital Trollhättan, Bromma Geriatric Hospital, Västmanland Hospital Västerås, Dalen Hospital and Lindesberg Hospital.

**Application for permission to pool variables with Riksstroke**

EFFECTS now has about one year left of inclusion. When we go through the variables we have collected, we notice that we have failed to include questions following a completed intravenous thrombolysis and thrombectomy. One alternative would be to go through all the patients' medical records, something that we are authorised to do, but it would be more effective to link this task with the quality register Riksstroke. The variables we require are:

1. Thrombolysis performed for stroke
2. Date of thrombolysis therapy
3. Thrombectomy or other catheter-based (endovascular) treatment for stroke
4. Date of thrombectomy

In addition, we also wish to have access to 4 variables prior to stroke (also taken from Riksstroke):

5. Need for assistance
6. Mobility
7. Toilet visits
8. Dressing

These variables form the basis of the algorithm that Riksstroke uses to assess the modified Ranking Scale. The purpose of linking these with the EFFECTS study is to examine how consistent the Riksstroke's algorithm is with the way we calculate the modified Ranking Scale in EFFECTS, namely the small modified Ranking Scale questionnaire (smRSq).

In order to facilitate the assessment of the study in the future, we have now updated the research plan with a version history – "Version history of the protocol". Previously, this version history has been documented in a separate document, but we believe that this increases transparency, updating the research plan to v 5.0.

**Application to send a priority questionnaire to participants in the study**

When we planned the EFFECTS study, we intended to include a patient representative in the steering committee. Unfortunately, we did not succeed in doing this. We believe that prior to the forthcoming study, it would be of great value to find out what stroke patients find important to research. Therefore, we would like to send out a questionnaire to anyone who participated and ask which issues should be prioritised in the future. We have referred to a study in the UK(1) where patients, relatives and staff are asked to state the 10 most important priorities. Based on this, we have created a questionnaire in which we now want the patients to rank the various topics. The questionnaire comprises one page and takes about five minutes to answer. We think that it should be send it out about 7 months into the study and be answered completely anonymously. We will not send out any reminders. Before we send out the survey, we check that the patient is alive.

We do not believe that they should be a significant burden for the individuals. The questionnaire will be returned in a prepaid envelope. The next page illustrates the questionnaire.

A fee of SEK 2,000 with the reference *Amendment 7 EFFECTS/Lundström* has been paid.

Erik Lundström
Chief Investigator EFFECTS

<u>Appendices:</u>
Copies of Resource Certificate for new centre
EFFECTS Protocol version 5 0 EU-nr_2011-006130-16

Reference


1. Pollock A, St George B, Fenton M, Firkins L. Top 10 Research Priorities Relating to Life after Stroke – Consensus from Stroke Survivors, Caregivers, and Health Professionals. Int J Stroke. 1 April 2014;9(3):313–20.

**Future research areas within stroke**

We would like to know the three areas that you feel are most important for us to research in the future. Rank the following areas from 1 to 3, where 1 is what you consider to be most important based on your situation, 2 is the second most important and 3 is the third most important area. You can send the questionnaire back to us in the prepaid envelope. Your answers will be treated completely confidentially and it will not be possible to see what answer you specifically have given. We will not send out any reminders.

Thank you for participating in the EFFECTS study,

Erik Lundström Chief
Investigator for EFFECTS
Associate Professor and Senior Consultant in Neurology

| **Your ranking** | **Research area** |
|---|---|
| | How can patients and relatives be helped to accept the long-term consequences of a stroke? |
| | How can patients be helped to recover from speech difficulties after a stroke? |
| | How can balance, walking and movement ability be improved after a stroke? |
| | How can the function of a person's arms and legs be improved/regained after a stroke? |
| | How can cognition (mental processes) be improved after a stroke? (Mental processes = brain function for processing information and using new knowledge. Functions include processes that require mental ability, such as attention, the ability to interpret, learning, memory, understanding, judgment and decision making.) |
| | How can vision problems be improved after a stroke? |
| | How can stroke patients and relatives be helped to handle speech problems after a stroke? |
| | How can patient self-confidence be improved after a stroke? |
| | How can fatigue be improved after a stroke? |
| | Are exercise and physical training programmes good for improving functional ability and quality of life after a stroke, and to avoid further stroke? |
| | Another area? ____________________________________ |

Clinical trials – Licences
Maria Lüttgen/is

**Decision**

Date: 8 August 2014 **EU no. 2011-006130-16**

Ref. 5.1-2014-43006

Dr Veronica Murray
Karolinska Institutet
Danderyd Hospital
182 88 Stockholm

**Permission for clinical pharmaceutical study**
*Clinical study of Oxatin (fluoxetine) (EFFECTS2012)*

You have applied for permission to carry out a clinical pharmaceutical study.

Pursuant to § 14 of the Swedish Pharmaceuticals Act (1992:859), the Swedish Medical Products Agency grants permission to carry out the clinical pharmaceutical study.

In accordance with LVFS 2011:19, a decision from the Ethical Review Board should be sent to the Swedish Medical Products Agency no later than 15 days from the day on which the sponsor receives notice of the decision.

On behalf of the Swedish Medical Products Agency

Maria Lüttgen
Specialist Physician

This decision document is not signed. This does not affect the validity of the decision.

Translated by
Språkservice
Reference: NKZ70
Malmö, Sweden: 20170920

Postal address: P.O. Box 26, SE-751 03 Uppsala, SWEDEN
Visiting address: Dag Hammarskjölds väg 42, Uppsala
Phone: +46 (0)18 17 46 00 Fax: +46 (0)18 54 85 66
Internet: www.lakemedelsverket.se E-mail: registrator@mpa.se

Translated by
Språkservice
Reference: NKZ70
Malmö, Sweden: 20170920
