# Supplementary material for: Update on the EFFECTS study of fluoxetine for stroke recovery: a randomised controlled trial in Sweden
Source: Trials. 2020 Feb 28;21:233. doi: 10.1186/s13063-020-4124-7 (PMC7048055; doi:10.1186/s13063-020-4124-7)
Supplement: Supplementary file 4 — Additional file 4. Overview of protocol versions in EFFECTS. [file 13063_2020_4124_MOESM4_ESM.docx]

Overview of protocol versions in EFFECTS

| **Version** | **Revision** | **Justification** |
| --- | --- | --- |
| Version 4.2 Date 2013-06-28  Version 4.3 Date 2013-09-17 Approval REC 2013-09-30  Approval MPA 2014-08-08 | Version 4.2 was the original version when EFFECTS applied to the Research Ethical Committee (REC) and the Medical Product Agency (MPA). Version 4.3 after request from the REC and MPA. | We received some questions from REC regarding consent form (minor formulations) and made subsequent changes in the Patient Consent Form (v2) and from MPA regarding pharmaceutical documentation. |
| Version 4.4  Date 2015-01-05 | No revision. Submitted to Medical Product Agency in Sweden | Co-chief Investigator Veronica Murray dies 2014-12-27 |
| Version 4.5 Date 2015-03-15; Amendment 1  Approval 2015-04-15 | Erik Lundström was appointed Chief Investigator and representant of the sponsor. Some changes in the Steering Committee.  Clarifying of the health economic study. | The need for organisation changes.  The health economic study was somewhat foggy. |
| Version 4.6  Date 2015-05-18;  Amendment 2  Approval 2015-06-10 | a) Changes in the patient consent form: The patient permits EFFECTS to obtain information from the central registry. We added “I also give my consent for information about being signed off sick, care-related consumption of resources and survival to be obtained from public registers. All data will be processed in anonymised form. Your personal data will be dealt with in accordance with the Swedish Data Protection Act. Danderyd Hospital is responsible for your personal data. You are entitled to receive an extract of your personal data once a year and can contact Eva Isaksson (tel. no. +46 (0)8 123 576 93) to obtain this.”  Some minor changes in the information about side effects in the consent.  b) Page 19 first paragraph changes from “more than 7 000 observed” to “up to 6 100 observed patients”  c) Page 21 paragraph 2.2.2. we added “Long-term data will also be retrieved from the Cause of Death Register and the National Patient Register, up to 3 years after inclusion of the last patient.”  d) Page 23, first paragraph, removal of the sentence “a printed eCRF, and a copy of all forms used.”  And we will add: “All forms will be possible to download from the trial website."  e) Page 30-31. The sentence “The total amount of capsules for six months is 186 capsules of fluoxetine 20mg and 186 capsules of matching placebo;” will be changed "The total amount of capsules for six months is 200 capsules of fluoxetine 20mg and 200 capsules of matching placebo;"  f) Page 35. Correction of the table: ”10.1. STUDY ASSESSMENT SCHEDULE.” We clarified the time interval.  g) Page 36, last sentence “The patient and relatives will receive a diary in which they are encouraged to record the date and nature of any adverse events.” is removed  h) Page 36. Remove “… will be sent or faxed to the coordinating center ...” and “... If no discharge form is received by 6 weeks the center will be prompted by fax or email to send the discharge form. If the patient is still in hospital the local research team will be asked ...”  And the following sentence is also removed:  “At these follow ups the GP or other responsible physician will be asked by the local EFFECTS-team about adverse events.”  Correction of the f/u: Face-to-face at 6 months, and central at 6 and 12 months. Removal of the possibility to have a web-based f/u.  i) Page 37: Sample size correction, correction from 6000 to 6100.  The following incorrect text is removed:  “The trial steering committee (TSC) will review the target sample size at the end of the feasibility phase and adjust this based on:  • Advice from the DMC  • Accruing data on  • the enrolment into specific pre-specified subgroups  • completeness of follow up  • distribution of mRS categories in the population of enrolled subjects (i.e. both treatment groups combined), overall and in specific patient categories (e.g. those with motor deficits, aphasia, etc)  For example, if the distribution of mRS is different to that anticipated, then the sample size might need to be increased. This approach has the advantage that such sample size adjustments can be made without reference to the accumulating blinded data and avoids the need for conditional power calculations which can be unreliable.”  j) Page 39. The following sentences will be removed:  “In this case the total population will be 1550, if however, trial eligibility has had to be changed we will report the 1500 from the main phase as main findings, and the 50 from the feasibility phase separately.”  Removal of the Fugl-Mayer scale and ANELT scale.  k) Page 40. Adjustment of the number of EQ5D-5L measurements during the main phase; a decrease from the measurement during the pilot phase of EQ5D-5L at 6 occasions (1 week, 4 weeks, 3 months, 6 months and 12 months) to measure it at 3 measurement points (inclusion, 6 and 12 months).  l) Page 43, Section 15.3.1, third paragraph.  We sharpen the writing of SUSARs. It must be reported through the help-line within 24 hours instead of by fax. The sentence now reads  “SUSAR should be reported to the Help-line (073- 663 74 44) within 24h.”  m) Clarifying that the centers only need to have the latest version of the protocol in their investigator site file.  Minor change in the CRF regarding MoCA. Removal of the Swedish personal security number.  n) Discharge form: Remove” Have there been changes in drug at baseline?”  o) Changes in Patient Consent form v 2015-05-18 v3, clarification of possible side effects of fluoxetine, as well as the request to use registry data.  The text now read: “I also agree that information on sick leave, health-related resource consumption and survival is obtained from public records. All data will be processed unidentified.  Your personal information is handled in accordance with the Personal Data Act. Responsible for your personal information is Danderyd Hospital. You may retrieve your personal information once a year and contact Eva Isaksson (tel. 08 123 576 93).” | a) We believe that registry date is a more appropriate and safer way to collect health economic data. At the same time, we do not need to burden the patients with questions.  b) Should read 6 100 (not 7 000)  d) We will not have any extra paper-CRF in the IB, The CRF can be downloaded via [www.effects.se](http://www.effects.se).  e) For simplicity, we will give the patient 100 + 100 capsules of the study medication.  f) Correction of table.  g) We will not have any patient diary  h) We want to simplify the process of the local center. To maintain security, we will encourage patient and relatives to call the local center to report. Our experience during the pilot phase is that this system works better, both patients and relatives find it easier to contact their local doctor or nurse.  The writing that we will have a special system with pre-enveloped envelopes and a web-based solution for patient and relative will be deleted.  We have reformulated the reading to match the follow-up performed (wrong writing in the protocol on this page), therefore we adjust the text to face-to-face follow-up at 6 months and supplementary central follow-up 6- and 12-months.  We will not have any web-based follow-up available to patients and relatives.  i) Minor adjustments. since our sister trial AFFINITY will include 1 600 patients (not 1500), and the total sum in the pooled number will read 6100.  j) We will recruit 1500 (not 1550) patients.  We will not use the Fugl-Mayer scale or ANELT (error writing)  k) We do not need 6 measurement points for quality of life.    l) Sharpening of the writing.  n) Discharge form: The previous motivation was a bit unclear, to clarify and simplify reformulate.  o) We believe that registry date is a more appropriate and safer way to collect health economic data. At the same time, we do not need to burden the patients with questions. |
| Version 4.7 Date 2015-11-12;  Amendment 3  Approval 2015-11-30 | Clarification of the health economic part of the trial, regarding EQ-5D and the use of VAS in the Stroke Impact Scale (SIS). We ensured that the VAS part of the EQ5D would be used in health economics. |  |
| Amendment 4 Approval 2016-06-14 | Clarification regarding the process of starting centers in EFFECTS. No protocol adjustments. |  |
| Version 4.8  Date 2015-12-21; Amendment 5  Approval 2017-01-04 | Page 24, exclusion criteria.  The company that manufactures fluoxetine has updated its Summary of Product Characteristics.  They now indicate that if metoprolol is used on indication heart failure, fluoxetine is contraindicated. EFFECTS Steering Committee and Safety Committee have concluded that this concerns serious heart failure that it may be clinically significant for more advanced heart failure (NYHA Grade III B – IV) and especially at higher doses and that co-administration of metoprolol and fluoxetine should be vigilant the interaction and early post-inclusion follow up the patient with clinical control including ECG.  Addition to exclusion criteria  “Fluoxetine is contra-indicated in combination with metoprolol used in cardiac failure New York Heart Association Grade III B and IV. At higher doses of metoprolol used on heart failure indication one should be vigilant of the interaction and early after enrollment monitor the patient with clinical monitoring including ECG.”  Page 26. Co-enrolment  Previously, we have written that participation in another CTIMP does not automatically exclude participation in EFFECTS, but it is important not to overload patients with studies. In the section on co-enrolment, we now refer to the TIMING study and add:  “It is allowed to co-enroll patients in EFFECTS and the TIMING-study. The intervention in TIMING is early vs delayed start of NOAC in patients with acute stroke and Atrial fibrillation. Thus, all patients would receive NOAC either <=4 days or > 5 days from the acute stroke.”  Page 29 Stopping Trial-treatment early.  We have observed that our protocol has not specified how long we recommend stop IMP for suspected adverse reactions and whether we will allow re-insertion of medicines after a long period of time. In the updated version, we have now clarified. We now add:  “We recommend coming off IMP for 14 days to see if the symptoms resolve. If they do, then ideally, they would restart to see if symptoms return. However, we recognize very few patients are prepared to do so. All stops (temporary and permanent) of the IMP must be registered in the e-CRF. There is not any limit for how long a temporary stop might be.”  Page 52, Protocol Amendments  In the protocol, we clarify that amendments relating to the addition of active centers in the study do not need to be sent to all centers as a protocol change. This is communicated in connection with major protocol changes as well as electronic via weekly newsletter and on the study's website (www.effects.se). | The company that manufactures fluoxetine has updated its Summary of Product Characteristics. We need to adopt to that.  Regarding co-enrollment, we specify in what extend we accept that.  We specify stop for suspected adverse reactions and whether we will allow re-insertion of study medication. |
| Version 4.9  Date 2017-03-24;  Amendment 6  Approval 2017-03-28 | Change of Principal Investigator at centre 3, Skövde hospital and centre 6, Karolinska University Hospital Huddinge.  Page 22. We will add:  The smRSq has been validated in English (Bruno 2010, 2011; Dennis 2012) but not in Swedish. We are planning to test the agreement of the Swedish small modified Rankin Scale questionnaire with face-to-face modified Rankin Scale. (Lundström manuscript synopsis 2017).  Synopsis of manuscript with preliminary title: Agreement of the Swedish small modified Rankin Scale questionnaire with face-to-face modified Rankin Scale.  The smRSq sends to the patients by the Trial Manager Assistant (TMA) at 6- and 12-month post randomisation. If the patient does not answer, the TMA contact the patient by phone and remind them to send in the questionnaire. If they have difficult to answer for themselves TMA helps them fill in the form by phone.  Statistics  Number of patients  The primary aim of the study is to evaluate whether the mRs-score measured by the smRSq differs from a mRS-score measured by a clinician. It has been defined that one step or more disparity in the mRs-score is a significant difference. A study of similar character has never been performed before and due to the nature of the study, an initial study, the sample size is not formulated in the guise of power, risk level, or clinical difference. The number of patients participating in the study is therefore primarily chosen for clinical reasons, not statistical, and 60 patients will be included in the study. In order to compensate for included patients not valid for efficacy analysis it is planned to enroll up to 65 patients in the study in order to have 60 patients valid for efficacy analysis. The attrition rate is estimated to be about 6%.  Statistical methods and data management  Statistical comparisons in order to test differences between dependent observations will be made by use of pair-wise Student's t-test for correlated means and statistical comparisons between two independent groups will be made by use of the Student’s t-test for uncorrelated means., after validation for normal distribution by use of the Shapiro Wilk test. The Pearson correlation coefficient will be used in order to test independence between variables. In addition to that descriptive statistics will be used to characterize the data. All analyses will be carried out by use of the SAS system (The SAS system for Windows 9.4., SAS Institute Inc, Cary, NC, USA.) and the 5% levels of significance will be considered. In the case of a statistically significant result the probability value (p-value) will be given. The results will be presented in a cross table. The proportion of full agreement will be given in percent and 95% Confidence Interval, as well as weighted and not weighted Kappa value. | Our primary outcome is the modified Rankin scale (mRS) measured with the small modified Rankins Scale (smRSq). The smRSq consists of five questions and can be conducted as survey or by telephone. smRSq is validated in English but not in Swedish. In our research plan, we have stated that we plan to do this in 2013. However, due to the fact that we have had to focus on other things (preparation of randomization systems, eCRF, inclusion of patients in the study), we have not completed the planned study.  Since it has been several years since we applied, we consider it important to clarify for the Regional Ethical Committee where we are in the matter.  We intend to investigate whether the survey we send at 6 and 12 months - the small modified Rankins Scale (smRSq) - gives similar results to a traditional assessment as face-to-face modified Rankin Scale. |
| Version 5.0  Date 2018-02-28; Amendment 7  Approval 2018-05-30 | We changed PI for centre 2 Karolinska University Hospital Solna, centre 14 Norrtälje Hospital, centre 19 Rehab Station Stockholm, centre 24 Stora Sköndal Neurological rehabilitation  Permission for pooling 8 variables from Riksstroke registry regarding IV thrombolysis and thrombectomy:  1.Thrombolysis performed for stroke, 2. Date of thrombolysis therapy, 3. Thrombectomy or other catheter-based (endovascular) treatment for stroke, 4. Date of thrombectomy. 5. Need for assistance, 6. Mobility  7. Toilet visits, and 8. Dressing.  Permission to send priority questionnaire on future research to participants in EFFECTS.  We have added a version history of the protocol. | We believe it is important to know the proportion of patients receiving IV thrombolysis and thrombectomy.  We want to compare the algorithm for smRSq and the variable used in Riksstroke registry.  It is important to know what patients think is important for future research. |
