# Supplementary material for: Update on the EFFECTS study of fluoxetine for stroke recovery: a randomised controlled trial in Sweden
Source: Trials. 2020 Feb 28;21:233. doi: 10.1186/s13063-020-4124-7 (PMC7048055; doi:10.1186/s13063-020-4124-7)
Supplement: Supplementary file 5 — Additional file 5. Consent to participate in EFFECTS and consent regarding handling notes and data management. [file 13063_2020_4124_MOESM5_ESM.docx]

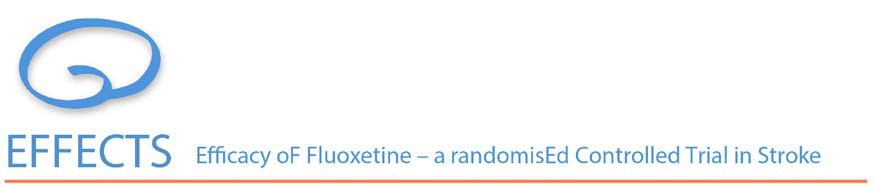


**A study of fluoxetine for stroke recovery**

• You have very recently had a stroke, and are therefore being asked to take part in a study.

• A stroke is caused by a blood clot or a cerebral haemorrhage that prevents the blood supply from reaching part of the brain.

• A common medication for depression and depression after a stroke, fluoxetine, has been shown in some smaller studies to improve recovery after a stroke by possibly helping the brain to repair the damage. Overall, more patients improved when they received fluoxetine, but the balance between the benefits and the risks involved with the treatment have not yet been established.

• Fluoxetine treatment involves side effects – one in ten patients experiences nausea, headaches or insomnia. As a general rule, these side effects pass within two weeks.

• Your doctor has made the assessment that fluoxetine can be combined with your other medications.

• We want to learn more about fluoxetine in order to improve stroke treatment.

• We would therefore like to ask you to participate in a study of fluoxetine in the case of stroke. Participation is entirely voluntary, and if you do not wish to take part this will not affect the other treatment you receive at the hospital in any way. You can also withdraw your participation at any time and without giving any reason, and this will not affect your other treatment at the hospital or after you are discharged.

• If you wish to take part in the study, you will receive study medication capsules containing either fluoxetine or an inactive substance, known as a placebo. This type of study is called a randomised controlled treatment study.

• The study medication consists of one capsule per day for a period of six months.

• Follow-ups will be carried out by telephone after one week, one month and three months, and one month after the end of the treatment. A repeat visit will take place at the hospital six months after the stroke. Follow-up by letter or telephone will also be carried out twelve months after joining the study.

• This information can be found in detail on pages 3-6.

• If you have any questions, we will be happy to provide more information.

Informed Consent Form 25/05/2018 version 4 Page 1 of 6

**CONSENT TO PARTICIPATE IN EFFECTS**

**and**

**CONSENT REGARDING HANDLING NOTES AND DATA MANAGEMENT**

**A study of fluoxetine for stroke revovery**

.................................................................. ...........................................................

(Patient’s name in capital letters) (Personal identity number)

I have read and received oral information, and I consent to take part in EFFECTS – a study of fluoxetine that may improve recovery in the case of a stroke.

I have had enough time to decide whether I want to be included in the study, and my questions have been answered.

I have been informed, and hereby give my consent, that relevant research leaders and any pharmaceutical control authorities may compare the data reported in the study with the data included in my patient notes.

I also give my consent for information about being signed off sick, care-related consumption of resources and survival to be obtained from public registers. All data will be processed in anonymised form.

Your personal data will be processed in accordance with the General Data Protection Regulation (GDPR 2016/679). Danderyd Hospital is responsible for your personal data. You are entitled to receive an extract of your personal data once a year. Please contact Eva Isaksson for additional information (08-123 576 93).

.................................................................. .........................................................

(Patient’s signature) (Today’s date)

.................................................................. ..........................................................

(Doctor’s signature) (Today’s date)

**CONSENT TO PARTICIPATE IN EFFECTS**

**and**

**CONSENT REGARDING HANDLING NOTES AND DATA MANAGEMENT**

**A study of fluoxetine for stroke recovery**

**If the patient is capable of making a decision but cannot give written consent.**

.................................................................. ..........................................................

(Name in capital letters) (Relationship to the patient)

We have received written and oral information about the study, and our questions have been answered. As my relative wishes to take part but is unable to give written consent, I give my approval for my relative to take part in the above study. We have also been informed, and hereby give our consent, that relevant research leaders and any pharmaceutical control authorities may compare the data about my relative reported in the study with the data included in his or her patient notes.

We also give our consent for information about being signed off sick, care-related consumption of resources and survival to be obtained from public registers. All data will be processed in anonymised form.

Your relative’s personal data will be processed in accordance with the General Data Protection Regulation (GDPR 2016/679). Danderyd Hospital is responsible for your relative’s personal data. Your relative is entitled to receive an extract of his or her personal data once a year. Please contact Eva Isaksson for additional information (08-123 576 93).

.................................................................. ..........................................................

(Signature) (Today’s date)

.................................................................. ..........................................................

(Doctor’s signature) (Today’s date)

**Detailed information about the EFFECTS study**

EFFECTS is an abbreviation of the name of our study. It stands for “**E**fficacy o**F F**luoxetine – a randomis**E**d **C**ontrolled **T**rial in **S**troke”. The Swedish name of the study is “Fastställande av effekt och säkerhet av fluoxetinbehandling vid stroke - en randomiserad placebo-kontrollerad studie av 1500 patienter”.

After a stroke, patients can be affected by many different problems: weakness or paralysis of the arms and legs, difficulties speaking, and disruption to memory and concentration. A stroke is often followed by fatigue.

The aim of EFFECTS is to investigate whether treatment with 20 mg of fluoxetine (one capsule daily) for a period of six months after an acute stroke can improve functional capacity.

Fluoxetine is now made by many different companies. It was initially marketed under the name Prozac®, and in Sweden the original preparation was called Fontex®. Fluoxetine has been used to treat depression for many years. Small studies have now also shown that the medication can improve recovery after a stroke

EFFECTS will include 1,500 patients at hospitals in Sweden. If the results show that fluoxetine is effective, this treatment could become part of routine healthcare after a stroke.

The reason why you have been asked to take part in this study is that you have recently had a stroke and you still have symptoms as a result of your stroke.

Participation in the study is entirely voluntary, and you can withdraw your participation at any time without it affecting your other care. Study data will be stored in a secure database that only the central people responsible for the study have access to. All information about you in the study will be stored under a specific study identification number and not under your usual personal identity number and name.

If you decide to take part, we would ask you to keep this information and for you, or a relative in consultation with you, to sign a consent form to take part in the study.

If you decide to take part, a nurse and the doctor providing treatment at your emergency hospital will record all significant information about your medical condition from your patient notes in the study journal. Your medical details will be added to a computer program which will randomly decide whether you will receive fluoxetine or a placebo. Half the patients in the study will receive a placebo, and half will receive fluoxetine. The placebo and the fluoxetine will be given in capsules. Neither you, your doctor nor any other care staff will know whether you are receiving fluoxetine or the placebo. If necessary, it will be possible to find out which you are receiving. Otherwise, this will not be revealed until after the end of the study.

Fluoxetine has few side effects, and is well tolerated as a general rule. The most common side effects usually pass within a few weeks of treatment. Examples of side effects include nausea, headaches and insomnia. If you develop any of these symptoms or anything else you do not recognise, please inform your responsible doctor or nurse at the emergency hospital, or your GP.

**Follow-up of study treatment**

One week and one month after you have started taking the study medication, the study group at the emergency hospital will get in touch to find out how you are feeling, whether you are taking one capsule of the study medication a day, and whether you have experienced any side effects. This will take 5-10 minutes.

After three months, the study nurse will call you again to find out how you are feeling and how the treatment is going. This will take around 15 minutes. Alternatively, this visit will be carried out at the hospital.

After six months, you will have a repeat visit at the clinic. You will also have to answer a questionnaire. The repeat visit will take around one hour.

One month after treatment has been completed, a study nurse will call to find out how you are feeling. This will take around 5-10 minutes.

Throughout the entire study period, you will have contact with your GP as usual, who will have been informed that you have had a stroke and are taking part in the study.

The last check-up within the study will take place 12 months after the start of the study, and you will answer the same questionnaire as after six months. This time, the questionnaire will be returned by post in a prepaid envelope. You can get help filling in the questionnaire by telephone. This will take 20 minutes.

**Possible benefits of taking part in the study**

If you have been part of the group treated with fluoxetine and the study shows that fluoxetine improves recovery after a stroke, you yourself may have benefited directly from taking part.

Some patients find it reassuring and helpful to take part in a research study and thereby have regular follow-ups.

If you suffer from depression after a stroke, it is more likely that this will be identified and treated. It is possible that the study treatment may prevent depression from occurring.

The study results which include your data will help doctors to treat future stroke patients better.

**Possible risks and disadvantages of taking part in the study**

Fluoxetine can produce side effects. However, as a general rule these are mild and pass. Serious side effects are possible, but these are rare.

Taking part in the research study will take up some of your time. Not all medications can be combined with fluoxetine.

**Results at the end of the study**

When the study has ended, we will write to notify you of the study results. This letter will tell you whether you received fluoxetine or the placebo.

**When happens if new information emerges during the study period?**

If new information becomes available that could affect the study or your personal participation, you will be contacted immediately.

**Does the study include any specific extra investigations?**

Yes, it includes being examined by your study doctor or study nurse and completing questionnaires.

**Who has reviewed the study?**

The Regional Research Ethics Committee at Karolinska Institutet and the Swedish Medical Products Agency.

**Possible side effects of fluoxetine**

Fluoxetine has been tested extensively in clinical studies among patients with depression. Patients suffering from depression after a stroke have also been treated.

**Side effects**

Fluoxetine treatment involves side effects – one in ten patients experiences nausea, headaches or insomnia. As a general rule, these side effects pass within two weeks. Other side effects may include loss of appetite, changes to taste perception, dryness of the mouth and weight loss. Patients sometimes experience sleep disruption and irritability. Fluoxetine can also increase the risk of bruising.

If you experience symptoms that you believe may be linked to the study medication, tell your study nurse so that he or she can contact your doctor responsible for the study.

| Responsible doctor | Responsible nurse |
| --- | --- |
| ………………………………………. | ………………………………………. |
| Tel.: | Tel.: |
