# Supplementary material for: Update on the EFFECTS study of fluoxetine for stroke recovery: a randomised controlled trial in Sweden
Source: Trials. 2020 Feb 28;21:233. doi: 10.1186/s13063-020-4124-7 (PMC7048055; doi:10.1186/s13063-020-4124-7)
Supplement: Supplementary file 6 — Additional file 6. List of centre and study personnel in EFFECTS. [file 13063_2020_4124_MOESM6_ESM.docx]

List of centre and study personnel in EFFECTS

Danderyd Hospital (192): Ann Charlotte Laska (PI), Elisabeth Änggårdh Rooth, Anna Grünfeldt, Eva Isaksson, Nina Greilert Norin, Hillevi Asplund. Karolinska University Hospital Solna (126): Bjarni Gudmundsson (PI), Malin Säflund, Maria Axelsson, Malin Bodin, Anna-Maria Parlatore. Skaraborg Hospital Skövde (102): Björn Cederin (PI), Eric Bertholds, Eva Åkerhage, Max Fantenberg. Hässleholm Hospital (49): Magnus Esbjörnsson (PI), Krzysztof Grodon, Erika Snygg, Anna Zenthio, Theres Strandberg. Uppsala University Hospital (77): Bernice Wiberg (PI), Erik Lundström, Oskar Fasth, Signild Åsberg, Semira Duzo, Solveig Bergqvist-Persson, Eva-Lis Lundberg, Gladys Gahongore, Käthe Ström, Rose-Marie Brundin, Malin Edén. Karolinska University Hospital Huddinge (15): Maria Lantz (PI), Ioanna Markaki, Caroline Bergmark, Gudrun Olsson-Skogman, Karin Söderquist, Kubra Talayhan, Magnus Hiis, Stefan Wrede. Mora Hospital (85): Jörg Teichert (PI), Solveig Östberg. Falu Hospital (22): Magnus Bergmann (PI), Mirja De Geer, Joakim Hambraeus, Monica Holst, Christina Nylen, Helen Luoma, Ingegerd Lönn, Sandra Johansson. Skaraborg Hospital Lidköping (12): Lennart Welin (PI), Anders Planck, Ingrid Roland, Sofia Wahll. Capio S:t Göran Hospital (77): Ulrika Löfmark (PI), Ahmad Ayad, Palpana Aikaterini, Christina Larsson, Gustaf Ahrenmark, Jan Mathé, Mezin Öthman, Marielle Anzén, Pirjo Perduv, Helena Thunberg. Visby Hospital (7): Sven-Erik Bysell (PI), Anna Vestberg-Bysell, Eva Smedberg. University Hospital of Umeå (15): Xiaolei Hu (PI), Per Wester, Britta Pettersson, Sara Korpela. Kristianstad Central Hospital (20): Axel Andersson (PI), Lena Eriksson. Norrtälje Hospital (9): Moa Gunnarsson (PI), Ann Engqvist, Hanna Hult, Annelie Fredlund, Linnea Nikander. Helsingborg Hospital (21): Pernilla Sandgren (PI), Camilla Werke, Petra Karlsson, Malin Karlsson. Skåne University Hospital Malmö (59): Eva Ask (PI), Martin Söderholm, Aniko Kuris, Elisabeth Poromaa, Natalie Montevert, Cecilia Johnsson. Halland Hospital Halmstad (74): Peter Thomasson-Sommer (PI), Miriam Morell-Larsen, Anders Funkquist, Lisbeth Andersson, Sofia Paulsson. Mälarsjukhuset Eskilstuna (36): Bo Danielsson (PI), Aleksander Saric, Anna-Karin Wärme, Christina Widhe-Qvist, Göran Pelang, Linnea Fransson, Åsa Byström, Malin Dalqvist. Rehab Station Stockholm (28): Liisa Hopia (PI), Sabahudin Bjelak, Ivan Sunara, Helen Lundberg, Rainer Gerdes, Leena Peltovuoma, Petra Engblom. Skåne University Hospital Lund (9): Andreas Arvidsson (PI), Irina Dragancea, Eva Engström, Lena Stankoska, Madeleine Rosen. Sundsvall Hospital (95): Fredrik Björck (PI), Vilhelm Sjögren, Anna Eelde, Anna Aronsson, Maria Kaldensjö. Sahlgrenska University Hospital (47): Anke Brederlau (PI), Arne Allardt, Anahita Nasouri, Annika Nordanstig, Cristina Gutierrez-Perez, Lukas Holmegaard, Maria Davidson, Mikael Jerndal, Margareta Ohlsson, Petra Redfors, Matilda Errind Arvgård, Stine Mathilde Hildal Fagerlind, Anna Bengtsson, Axel Hallingbäck, Anna Jacobson, Cecilia Damm, Lena Rosen. Högsbo Rehabilitation Hospital (2): Trandur Ulfarsson (PI), Marie Gustafsson. Stora Sköndal Neurologic Rehabilitation Clinic (16): Mehran Taklif (PI), Anna Sjöström, Ann-Kristina Hamrin, Åsa Samuelsson, Judith Treuter, Lena Lindvall. Östersund Hospital (95): Magnus Gibson (PI), Joachim Ögren, Linn Tander, Linda Wiklund. Alingsås Hospital (73): Brita Eklund (PI), Kjersti Hellqvist, Anna Lindh, Ida Abrahamsson, Karin Sjöström de Andrade, Maria Ekholm. Ängelholm Hospital (40): Björn Hedström (PI), Antonia Boldt-Christmas, Benny Ranebjer, Indre Valanciene, Annelie Rasmusson, Jenny Nilsson. Stockholm Sjukhem Neurologic Rehabilitation Center (29): Ellinore Richardsson, Jonas Björling, Helena Ekman, Pia Storbacka, Tarja Thiang. Örebro University Hospital (10): Daniel Merrick (PI), Anders Häggström, Lisa Ahlberg Jangentorp, Carina Ragnemyr. Northern Älvsborg County Hospital (7): Per Broman (PI), Ioanna Dagiasi, Marita Olofsson, Vivi-Anna Dahy. Stockholms Sjukhem Geriatrics (1): Max Mademyr-Larsson (PI), Anuja Withana, Carina Thoren, Haben Tekesteokbai. Västmanlands Hospital Västerås (42): Andreas Ranhem (PI), Ammar Yousif, Hannes Frejd, Per Lenngren, Catharina Holmberg, Joanna Nilsson Ryding, Linda Nyren, Maria Sellin, Sara Östring-Jalonen. Dalen Hospital (3): Camilla Ronnheden (PI), Dag Salej, Viktoria Westerlund, Ann-Christine Alvin, Catharina Ryberg, Helena Larsson, Maria Ljunggren. Lindesbergs Hospital (17): Martin Johansson (PI), Carl Bring, Kent Karlsson, Anna Wendelstam. Hudiksvalls Hospital (22): Anette Onkenhout (PI), Per-Gunnar Wiklund, Adam Lyren, Amanda Engberg, Moa Nordström, Sandra Åström, Viktoria Hjern.
