# Supplementary material for: Update on the EFFECTS study of fluoxetine for stroke recovery: a randomised controlled trial in Sweden
Source: Trials. 2020 Feb 28;21:233. doi: 10.1186/s13063-020-4124-7 (PMC7048055; doi:10.1186/s13063-020-4124-7)
Supplement: Supplementary file 7 — Additional file 7. Monitoring Plan. [file 13063_2020_4124_MOESM7_ESM.docx]

Karolinska Trial Alliance

Establishing the effect(s) and safety of fluoxetine initiated in the acute phase of stroke

EudraCT number: 2011-006130-16

MONITORING PLAN

(updating of previous version signed on 01/03/2016)

Sponsor: Karolinska Institutet, Danderyd Hospital

Department of Clinical Sciences, Stockholm

Representative for sponsor: Erik Lundström

Chief Investigator: Erik Lundström, MD, PhD

Department of Neurology, Building R#:04

Karolinska University Hospital Solna

171 76 Stockholm

[Erik.lundstrom@ki.se](mailto:Erik.lundstrom@ki.se) tel: +46 (0)8-5177 4697, +46 (0)70-767 7411

Trial locations: Approx. 35-40 clinics in Sweden

Trial Manager: Eva Isaksson, RN

Danderyd Hospital, Dept of Medicine

182 88 Stockholm

[Eva.isaksson@ds.se](mailto:Eva.isaksson@ds.se) +46 (0)8-1235 7693, tel: +46 (0)70-340 4892

Monitor: Ingalill Reinholdsson (contact person)

Karolinska Trial Alliance, KTA Support

Norra Stationsgatan 67

171 76 Stockholm

[Inga-lill.reinholdsson@karolinska.se](mailto:Inga-lill.reinholdsson@karolinska.se) tel: +46 (0)70-002 1375

20/01/2017, version 3 Page 1 of 5

Terese Brunsell

Karolinska Trial Alliance, KTA Support

Norra Stationsgatan 67

171 76 Stockholm

[terese.brunsell@karolinska.se](mailto:terese.brunsell@karolinska.se) tel: +46 (0)72-580 2945

Maria Persson

Karolinska Trial Alliance, KTA Support

Norra Stationsgatan 67

171 76 Stockholm

[maria.h.persson@karolinska.se](mailto:maria.h.persson@karolinska.se) tel: +46 (0)72-599 1259

1. STUDY TITLE

Establishing the effect(s) and safety of fluoxetine initiated in the acute phase of stroke.

A academic-driven randomised, placebo-controlled study to investigate whether treatment with 1 20mg capsule of fluoxetine compared with 1 placebo capsule a day for 6 months following an acute stroke can improve the patient’s functional ability.

2. TRIAL CENTRES

Approx. 35-40 clinics in Sweden.

3. PATIENTS

Number of patients in the study: 1,500 patients

Treatment time per patient: 6 months

Total study time/patient: 12 months

Timetable:

Pilot study (50 patients) 2014-2015

Main study (1,450 patients) 2015-2019

4. TERMS FOR MONITORING

KTA conducts monitoring according to ICH-GCP, the Declaration of Helsinki, the protocol and the monitoring plan.

20/01/2017, version 3 Page 2 of 5

5. RISK ASSESSMENT

Study design: A randomised, placebo-controlled, double-blind study.

Is there anything in the design that imposes extra requirements on patient safety and/or the study results?

Description: Multi-centre study with approx. 30-40 clinics in Sweden **Yes** No

Patient group:

With acute illness

Description: Patients being treated after acute stroke **Yes** No

Trial drug: Fluoxetine

Approved **Yes** No N/A

Known side effects **Yes** No N/A

Much time required to count the medication Yes **No** N/A

Study familiarity of the trial centres:

All staff involved will be offered GCP training within the framework of the study.

CRF

eCRF was implemented in autumn 2015 Yes No

Paper CRF was used previously.

6. MONITORING

The purpose of the monitoring is to verify that the protocol is being followed, and that the patients’ well-being and safety is being safeguarded. Through total monitoring of 10% of the patients, this ensures that the data collected by the study maintains a high quality.

The first monitoring visit will be made after the first patient/centre has had their 3-month appointment, or as agreed with the Trial Manager. Then 10% of the patients/centres will be monitored. The monitor contacts the study centre to set a date and time for the monitoring visit.

Scope of the source data verification

Source data verification will be done according to the table below.

20/01/2017, version 3 Page 3 of 5

| Scope | Parameter | Comment |
| --- | --- | --- |
| First patient/centre and 10% of the patients/centres | Inclusion and exclusion criteria | Verify that the correct patient is included. |
| First patient/centre and 10% of the patients/centres | NIHSS, MADRS, DSM-IV, MoCA, NGTA, EQSD-5L | Verify that all forms are filled out. |
| First patient/centre and 10% of the patients/centres | Randomisation | Verify that the randomisation form is complete and that the correct information is transferred to the randomisation system and that the correct randomisation number is assigned to the patient. |
| First patient/centre and 10% of the patients/centres | Trial drug/Drug accountability | Verify in the records and Inventory and dispensing log that drug container 1 and 2 have been given to the patient. Verify the number of tablets/containers that have been returned to the clinic. No stock control. |
| First patient/centre and 10% of the patients/centres | Other drugs | Check whether non-authorised drugs have been prescribed. |
| First patient/centre and 10% of the patients/centres | AE and SAE* | Verify that AE has been noted, assessed and that any SAEs are reported. |
| First patient/centre and 10% of the patients/centres | Phone and clinic appointments | Check that phone and clinic appointments take place in accordance with the protocol and that the CRF modules are filled out. |
| First patient/centre and 10% of the patients/centres | Informed consent | Correct version and correctly dated and signed. Verify against records. |

*Definitions of AE, SAE are in the study protocol.

Essential documents

Check that all essential documents are in the investigator file and that logs are completed at the respective centre.

Follow-up of monitoring

After each monitoring visit, a monitoring report is sent to the investigator at each site and to the Chief Investigator and Trial Manager. The monitoring report contains a description of the findings during the visit and information on what needs to be addressed before the next monitoring visit.

20/01/2017, version 3 Page 4 of 5

As representative for the sponsor of this study, I hereby approve the monitoring plan.

10/02/2017 [signature]

Date Erik Lundström

Chief Investigator EFFECTS

Representative for Karolinska Trial Alliance

20/01/2017 [signature]

Date Ingalill Reinholdsson

Monitor

20/01/2017, version 3 Page 5 of 5
