# Supplementary material for: Update on the EFFECTS study of fluoxetine for stroke recovery: a randomised controlled trial in Sweden
Source: Trials. 2020 Feb 28;21:233. doi: 10.1186/s13063-020-4124-7 (PMC7048055; doi:10.1186/s13063-020-4124-7)
Supplement: Supplementary file 8 — Additional file 8. Data Management Plan for EFFECTS. [file 13063_2020_4124_MOESM8_ESM.pdf]

EDC Scandinavia AB

Data Management Plan for EFFECTS

DMP

also referred to as the Data Handling Protocol

Establishing the effect(s) and safety of fluoxetine initiated in the acute phase of stroke

EudraCT Number 2011-006130-16

Version Number<br><br>1.0	Approval Date:<br><br>2016-10-05
Superseded Version Number and Date:	

Disclaimer

When using this document please ensure that the version you are using is the most up to date by contacting EDC Scandinavia to confirm the current version.

Out of date documents must not be relied upon.

SIGNATURE PAGE

AUTHOR:
The author's signature indicates that this document was written for the named system to meet the EDC Scandinavia AB (EDC Scandinavia) quality standards for a Data Management Plan for EFFECTS EudraCT Number 2011-006130-16.

Krister Kristianson, CTO<br>EDC Scandinavia AB		Date 2016-10-05

APPROVAL SIGNATURE

The signatures indicate that this document was approved as the Data Management Plan for EFFECTS EudraCT Number 2011-006130-16.

Ingalill Reinholdsson, monitor<br>Karolinska Trial Alliance		Date 2016-10-06

Erik Lundström, Chief Investigator<br>EFFECTS		Date 2016-10-17

History

Revision	Comments	Print date/sign
1.0	New	

Contents page

	Signature page	2
1.0	Background	4
2.0	Study personnel	4
3.0	Study objectives and design	4
4.0	Timescales and key activities	5
5.0	Procedures	5
5.1	Basic Terms and Concepts	5
5.2	Data Management Process	5
5.3	Data Management Databases	6
5.4	Data entry & data processing	7
6.0	Data Validation	7
7.0	Discrepancy Management	8
8.0	Medical Coding	8
9.0	Database Locking	8
10.0	Archiving	8
11.0	User Training	10
12.0	References	10
13.0	List of abbreviations and acronyms	11

1.0 BACKGROUND

This data management plan is intended to describe the activities and responsibilities for the data management. It also provide guidance for managing data and ensuring all trial data are collected and verified in the appropriate manner to certify that data are recorded correctly and that a validated clean data file is delivered to the statistician of EFFECTS.

2.0 STUDY PERSONNEL

The task is divided between KI, KTA and EDC Scandinavia AB personnel and the three companies will make sure that qualified staff is available for the task.

The responsibilities for the following Data Management (DM) activities are divided between the sites, KI, KTA and EDC Scandinavia as follows:

Database design	EDC Scandinavia
e-CRF design	EDC Scandinavia, KI and KTA
Server management	EDC Scandinavia
Data Collection	Site and KI
Data manger	EDC Scandinavia
CRF annotation	Site, KI and KTA
Data entry	Site, KI and KTA (only central 6 and 12 months)
Monitoring	KTA
SDV	KTA
Issue DCF's	KI & KTA
Resolve DCF's	KI & KTA
Medical Coding	KI
Data validation	EDC Scandinavia and KI
Discrepancy Management	Site, KI & KTA
Database lock	EDC Scandinavia

3.0 STUDY OBJECTIVES AND DESIGN

Several small trials have suggested that fluoxetine may improve neurological recovery from stroke. EFFECTS is a investigator-led, multicentre, parallel group, randomised, placebo-controlled trial which aim to determine whether the routine administration of fluoxetine (20mg daily) for six months after an acute stroke improve patients' functional outcome.

The trials include patients $\geq$ 18 years old with a clinical diagnosis of stroke, persisting focal neurological deficits at randomisation between two and 15 days after stroke onset. Patients are randomised centrally via web based randomisation systems using a common minimisation algorithm. Patients are allocated Fluoxetine 20mg once daily or matching placebo capsules for six months. The primary outcome measure is the modified Rankin scale (mRS) at

six months. Secondary outcomes include: Living circumstances, the Stroke Impact Scale, EuroQol (EQ5D-5L), the vitality subscale of the Short-Form 36, diagnosis of depression, adherence to medication, adverse events, and resource use. These are collected at 6 and 12 months.

Trial registration:
EudraCT Number 2011-006130-16 (registered in 2011)
ISRCTN13020412 (19/12/2014)
Clinicaltrials.gov number NCT02683213 (02/02/2016)

4.0 TIMESCALES AND KEY ACTIVITIES

Activity	Responsible	Planned timeline
SDV & monitoring	KTA	Ongoing
Medical coding of AE's	KI	Ongoing
Data clean up	Site, KI, statistician & KTA	Ongoing
All CRF's signed by PI	PI	Ongoing
Final preparation for clean file: Final review and Database freeze	KI, statistician and Chief Investigator, EDC Scandinavia & KTA	2019
Clean file meeting	Chief Investigator, statistician, EDC Scandianavia, KI & KTA	2019
Final site activities	KTA	2019
Final data review	EDC Scandinavia	2019
Database lock	EDC Scandinavia	2019
Data export to statistician	EDC Scandinavia	2019

5.0 PROCEDURES

5.1 Basic Terms and Concepts

5.1.1 Source data

The Source Data List defines the source documents and the source data in this study. This can vary between sites.

5.2 Data Management Process

The data management process will involve evaluating data collected using OpenClinica data collection tools and queries in MS ACCESS (Microsoft Redmond, WA) to allow statistical analysis to be conducted. The e-CRF electronic documents are designed to record all of the protocol-required information to be reported to the statistician on each Trial participant.

5.3 Data Management Database

During the installation of OpenClinica® (manufactured by OpenClinica LLC

Waltham MA, USA) system a PostgreSQL (PSQL) database is set-up that is validated together with the other OpenClinica® installations. All data management is performed via the OpenClinica® interface and no activities are performed directly in the PostgreSQL (PLSQL) database. The purpose of the management functions are to:

- Verify data integrity
- Raising data queries
- Traceability of data corrections
- Code AE terms
- Ensure the security of the database and Clinical Trial data

5.4 Data entry & data processing.

5.4.1 Data entry

1- The required information is filled in on the data entry screens by the clinic staff. Many fields are mandatory and the screens are dynamic, meaning that some field's behaviors are depending on entries in the preceding field.
2- Following data entry, the study monitor should do a complete Source Data Verification (SDV) according to the monitoring plan, and any corrections or additions should be made by the site in the database.
3- When the e-CRF has been marked "SDV completed", the Data Manager at EDC Scandinavia will start a review for any missing data, incomplete fields or data outside normal ranges. Other inconsistencies will also be looked at. Protocol violators will be looked for as defined in the protocol.
4- If any discrepancies are raised at this stage, a query is entered by the Data manager in the Discrepancies and Notes (DNOTES) system in OpenClinica®. The query is both in the DNOTES system and in the audit trail for review by the regulatory inspectors e.g. Läkemedelsverket. In DNOTES any change after the CRF data is marked "COMPLETE" is stored with user ID's and further details to follow the change accurately.
5- Any mandatory missing data must be explained in DNOTES. Missing data are represented by a blank field. At the end of the study, any missing data will be listed by patient with an explanation.
6- The final electronic signature will be made by the investigator once all corrections have been done, and no open questions remain as DCF's or in the DNOTES system.
7- It is essential that there is a close cooperation between the KI, KTA monitors and the EDC Scandinavia staff on the DCF status and that EDC Scandinavia is notified when they have been resolved.

6. DATA VALIDATION

The EDC Scandinavia Trial Data Review (TDR), a MS ACCESS based system testing each CRF system will be used in order to identify any discrepancies in the entered data, which are embedded in the database, to ensure data validity. However, only data that has undergone SDV is included. Ongoing quality control of data processing is undertaken at regular intervals during the course of the study according to the monitoring plan. At the end of the study the DNOTES system will

also be loaded into TDR to make sure there are no unresolved questions remaining.

7. DISCREPANCY MANAGEMENT

Discrepancy management includes reviewing discrepancies, investigating the reason, and resolving them with documentary proof or declaring them as irresolvable. Discrepancy management helps in cleaning the data and gathers enough evidence for the deviations observed in data.

Based on the types identified, discrepancies are flagged for the site and monitor on the DCF for clarification or closed in-house by the monitors to the site via the OpenClinica system. The most common Self Evident Corrections (SEC) are obvious spelling errors. For discrepancies that require clarifications from the investigator, DCFs will be sent to the site by the monitor. When a resolution is provided by the site staff, they will update the database.

The CDM team reviews all discrepancies at regular intervals to ensure that they have been resolved. The resolved data discrepancies are recorded as 'closed'. This means that those validation failures are no longer considered to be active, and future data validation attempts on the same data will not create a discrepancy for same data point.

8. MEDICAL CODING

Adverse events occurring and coded World Health Organization ICD codes according to the Study Protocol and Statistical Analysis Plan. If information of the ongoing medication is needed, World Health Organization ATC code is used for coding.

9. DATABASE LOCKING

After the final data has been checked off and all DCF's have been resolved a final data validation is run. If there are no remaining discrepancies, the datasets are finalized in consultation with the statistician. All data management activities should have been completed prior to database lock. To ensure this, a pre-lock checklist is used and completion of all activities is confirmed. This is done as the database cannot be changed in any manner after locking. Once the approval for locking is obtained from all stakeholders, the database is locked and clean data is extracted for statistical analysis according to the statisticians needs, usually as Excel or tab delimited text. Generally, no modification in the database is possible, but in case of a critical issue or for other important operational reasons, privileged users can modify the data even after the database is locked. This, however, requires proper documentation and an audit trail has to be maintained with sufficient justification for updating the locked database. Data extraction is done from the final database after locking.

10. ARCHIVING

The Archiving of the study will be done by the sponsor. All study data will be delivered to the sponsor in Operational Data Model (ODM) 1.3 Clinical Data

Interchange Standards Consortium (CDISC) format on different digital media such as USB stick and DVD to guarantee the readability during the retention period considering the longevity of such media. All CRF patient data can also be delivered to the sponsor as printable documents in PDF format containing the audit trail with signature information if requested.

11.0 USER TRAINING FOR DATA ENTRY

The user training for OpenClinica is a 3 hour session where all aspects of data entry and management from a data entry user point of view are demonstrated in either a lecture or an online session where the users can also practice on their own computers. A demo video is first shown, followed by a step by step demonstration on a copy of the production database. To further improve on the user training, a copy of the production database is available on a specific server at EDC Scandinavia (https://www.edcecrf.com:8443/OpenClinica). This will allow for direct on-line access and all users are set up with their own user ID and passwords. This database makes it possible to practice all aspects of data entry and editing in a "live" environment. A specific emphasis is focused on Good Data Entry Practices. Upon request, a special session can be set up for monitors training, demonstrating the use of the SDV and Notes & Discrepancy system in greater detail.

Procedure:

1) The sponsor or KTA sends a request for a new user ID to be created
2) An account is created by the Trial Management Assistant
3) A session is set up with all users to be trained according to above procedures
4) At the training session a user manual will be distributed explaining in detail all the steps for data entry and editing. This user manual is study specific.
5) Access to the production database will be given when the "User authorization form is signed.

12.0 REFERENCES

ACDM Guidelines to facilitate the production of a data handling protocol
International Conference of Harmonization (ICH) of Good Clinical Practice.
European Clinical Research Infrastructures Network - GCP-compliant data management in multinational clinical trials.
ICH Topic E 6 (R1) Guideline for Good Clinical Practice CPMP/ICH/135/95 July 2002.

13.0 LIST OF ABBREVIATIONS AND ACRONYMS

Abbreviation, acronym or specialist term	Explanation
ACDM	Association For Clinical Data Management
AE	Adverse Event
ATC	WHO coding of drugs
CDISC	Clinical Data Interchange Standards Consortium
CDASH	Clinical Data Acquisition Standards Harmonization
CRF, e-CRF	Case Report Form (paper) , electronic Case Report Form
CDM	Clinical Data Management
DCF	Data Correction Form
DMC	Data Monitoring Committee
DNOTES	Discrepancies and Notifications
DRP	Data Review Plan
DVP	Data Validation Plan
EDC	Electronic Data Capture
GCP	Good Clinical Practice
EDC Scandinavia	EDC Scandinavia AB
FPI	First patient in
ICH	International Conference on Harmonisation
KI	Karolinska Institutet (sponsor)
KTA	Karolinska Trial Alliance
LPI	Last patient in
LPO	Last patient out
LVFS	Läkemedelsverkets författningssamling
ODM	Operational Data Model
PI	Principal Investigator, referring to the local center
PSQL	PostgreSQL
QA	Quality Assurance

**Abbreviation, acronym or specialist term**	**Explanation**
QC	Quality Control
SAE	Serious Adverse Event
SEC	Self Evident Corrections
SDV	Source Data Verification
SOP	Standard Operating Procedure
SUSAR	Suspected Unexpected Serious Adverse Reaction
TDR	Trial Data Review System
USB	Memory Stick
