# Supplementary material for: Update on the EFFECTS study of fluoxetine for stroke recovery: a randomised controlled trial in Sweden
Source: Trials. 2020 Feb 28;21:233. doi: 10.1186/s13063-020-4124-7 (PMC7048055; doi:10.1186/s13063-020-4124-7)
Supplement: Supplementary file 9 — Additional file 9. Data Review Plan (DRP) for EFFECTS. [file 13063_2020_4124_MOESM9_ESM.pdf]

Data Review Plan (DRP) for EFFECTS

also referred to as the Data Validation Plan (DVP)

Establishing the effect(s) and safety of fluoxetine initiated in the acute phase of stroke

EudraCT Number 2011-006130-16

Document number: DRP-01	Effective Date:<br>2017-11-22
Version Number & Date<br>Ver. 1.0 207-11-22	Approval Date:<br>2017-11-22

Disclaimer

When using this document please ensure that the version you are using is the most up to date by contacting EDC Scandinavia AB to confirm the current version.

Out of date documents must not be relied upon.

SIGNATURE PAGE

AUTHOR:

The author's signature indicates that this document was written for the named system to meet the EDC Scandinavia AB (EDC) quality standards for a Data Review Plan for EFFECTS EudraCT Number 2011-006130-16.

Krister Kristianson<br>---------------------------------------<br>Krister Kristianson<br>Chief Technical Officer,<br>EDC Scandinavia AB	KK	Date 2017-11-22

APPROVAL SIGNATURE

The signatures indicate that this document was approved as the Data Review Plan for protocol EFFECTS EudraCT Number 2011-006130-16.

Approved ~~by~~ KTA

Ingalill Reinholdsson<br>---------------------------------------<br>Ingalill Reinholdsson, monitor<br>Karolinska Trial Alliance	IR	Date 2017-11-23

Approved by KI:

---------------------------------------<br>Erik Lundström, Chief Investigator<br>EFFECTS	EL	Date 2017-11-22

History

Revision	Comments	Print date/sign
1.0	New	2017-11-22 /Krister Kristianson

I. Overview

This Data Review Plan has been designed for the EFFECTS Protocol, a study being conducted to study Establishing the effect(s) and safety of fluoxetine initiated in the acute phase of stroke

Protocol EFFECTS is being conducted at 35 sites in Sweden. This plan describes the rationale and methods to be used to examine the databases for this study in order to ensure that the data are accurate, complete, and logically consistent before closing the study.

The need for a documented, protocol-specific data review plan stems from the flexibility afforded by the OpenClinica® data management tools, and the anticipated efficiency that can be achieved by having multiple reviewers. Data is entered into the database on an ongoing basis throughout the study, and can be reviewed by authorized individuals simultaneously. It is therefore important to systematize the review process, assign responsibilities for each step, and devise a means to communicate the progress of each party involved. This plan defines the basic sets of queries to be performed and how OpenClinica® allows us to define completion of "milestones" along the way. Additional queries may be conducted as the need arises.

By establishing a fairly comprehensive query strategy outlined by this document, potential discrepancies and clinical issues within the database will be addressed as thoroughly as possible. When all discrepancies and reviewer questions have been resolved, the review by protocol will be complete. This will allow for auditing to take place, and locking of the database for statistical analysis.

II. Concepts Addressed by the Data Review Plan

During the data capture and data entry process, simple errors or omissions may occur and medical issues may be overlooked, such as potential adverse events or protocol violations. It is the clinical team's challenge to uncover these discrepancies from a complex database by considering some basic questions:

- Are the data complete and accurate?
- Are the data internally consistent?
- Was the protocol followed?
- Are adverse events reported correctly?

Specific queries to answer these questions are designed for each type of data captured (laboratory, clinical, demographic, etc.), and are intended to be performed at different stages of data entry. The stages are broken down to achieve defined milestones, which are discussed below (IIIC).

III. General Methods

A. The OpenClinica® Database

Investigators participating in Protocol EFFECTS record the study data in e-CRF's provided by EDC using the OpenClinica® system Community version by OpenClinica LLC, MA, USA. Data entry is done by the investigators or the Clinical Study Coordinators at the clinic. In most cases the data is entered directly during the examination of the patient without using any paper source. The clinic is requested by law to enter some of the data into the clinic's Electronic Medical Records system as well.

Once in the database, data views can be queried using tools in the OpenClinica® system. The database can be accessed by authorized users from any computer connected to the internet. Changes to the database can only be made by users with and adequate role to allow data entry and change. The final CRF's are to be electronically signed by the investigator.

B. OpenClinica® Data Review and Correction Tools

The following descriptions of OpenClinica® Clinical Trials Applications System tools have been excerpted from the OpenClinica® Operations Manual, with some modification according to methods to be implemented for EFFECTS.

Data Management and Trial Data Review Systems

The Data Management System (DM) is a data review application in the OpenClinica® Clinical Trial System as well as a separate system in a SQL database. EDC staff can use standard queries and

reports to review data, or they can create their own ad hoc queries. Trial Data Review (TDR) is a supplementary data review application, offering additional programmed queries and customized views. The programmed queries in TDR and DM are provided due to the complexity of these particular queries, since no programing expertise is required to use the applications.

Notes & Discrepancy System (NDS)

When EDC staff use the DM and TDR, they may find some data discrepancies or have some medical questions that they wish to communicate to the study site. NDS provides the monitoring staff with an electronic mechanism for generating and tracking communications with the site with respect to such questions. All stages, from initiating a discrepancy question for the site, to receipt of a response, to verification of any resultant data revisions in the database can be tracked in NDS. NDS can be assigned to any user with a data entry role in order to communicate questions to the site, the central EFFECTS coordinating center or via the KTA CRA (Monitor), and record the response prior to any change to the database.

Example of the NDS Overview Statistics:

	Query	Failed Validation Check	Reason for Change	Annotation	Total
New	6	90	--	--	96
Updated	12	22	--	--	34
Resolution Proposed	4	24	--	--	28
Closed	212	87	4	13	316
Not Applicable	--	--	599	2392	2991
Total	234	223	603	2405	3465

C. Methods of Data Review

Data review is a shared task between the EFFECTS coordinating centre ~~office~~, KTA and EDC Scandinavia AB's personnel, under the assumption that each discipline will execute their review responsibilities completely and in a timely fashion. The KTA main focus during the review is on Source Data Verification (SDV) on 10% of the patients, and other issues discovered during their visits to the site according to the monitoring plan. The review strategy should also consider the degree or depth of review appropriate to different types of data and the likelihood that a review question will lead to a meaningful response and database change for data critical to the program.

1. EDC Scandinavia AB's review

EDC review is undertaken continuously during the entire review process for a study. It involves use of the NDS and TDR tools as well as ad-hoc queries run by the EDC team.

- **Adverse Events Reporting**

  Adverse Events Reporting is one of the initial functions a data reviewer will undertake in her/his daily data review routine. The data reviewer will review Adverse Events in accordance with the EFFECTS coordinating center, KTA and EDC's policies & procedures.

- **Discrepancy Tracking**

  The data reviewers will use data review tools to identify discrepancies in accordance with EFFECTS coordinating center, KTA and EDC's, Data Review procedures

  Queries

  The data reviewer will run the standard and protocol-specific queries on all data to locate any data questions which may be present. If a discrepancy is noted, the reviewer will query the site via the Notes & Discrepancy Tracking System or via e-mail. Queries can also be entered directly in the NDS system and assign to any authorized user of the system.

Monitoring Reports and Queries

In addition to the Global Review, which is intended to search for data discrepancies or inconsistencies, other monitoring queries will be performed on an ongoing basis by the designated data review person at the EFFECTS monitoring center and the CRA at KTA in order to track the progress of the studies and any trends in the data while still blinded. These will help uncover some possible safety concerns or procedural concerns within or across study sites if more than one site is entering patients.

Investigator Certification

After data entry review is complete, the finalization of the Case Report Form (CRF) is indicated by a green checkmark. The CRF is now ready for investigator review. The investigator will review the CRF to confirm that the data are representative of the source documents and that any additional comments or data revisions verified by the Investigator are documented in the site specific study file.

- If a data point is incorrect, the investigator may make the changes directly on the CRF. Upon resolution and/or approval, the next bullet is then applicable.

- If the data are correct, the Investigator will sign the CRF electronically by providing his/her userid + password and the CRF will get a stamp indicating that it is final.

<u>Medical Review by Protocol</u>

Medical Review by Protocol is part of the frozen file process described by EDC Scandinavia AB's Standards.

E. Tracking of Data Review

A checklist is provided electronically to track each patient's review and the progress toward each milestone. The checklist will document for a patient that each query was performed, that all encoding was completed, and that all Discrepancy Notifications Forms were satisfactorily resolved.

IV. Specific Methods: Query Sets

<u>QUERY SET</u>

This query set should be executed as soon as new patients have been entered. In the case of this rather long study execution should await all patient data to be complete and the final contact has been documented.

1. Determine which data have been marked "Data entry completed " by using the N&D statistics and any non-resolved N&D

2. In the Notes & Discrepancy System, search for "All Open" discrepancies by allocation number.
    - If an annotation addresses the discrepancy adequately, then close the discrepancy.

1. Data Review Tables and Queries
   The TDR application contains various view tables reflecting data from the collector modules, and standard and protocol-specific queries. Queries to be performed are listed in the tables on the following pages. Issue new discrepancies according to your observations, and follow the discrepancies through to closure. This may include updating the database, and verifying the data change. Refer to annotations to make sure items are not already addressed.

2. EDC will provide the Chief Investigator with appropriate data for allowing submission to ClinicalTrials.gov

QUERY SET 1:

Question	Query Tool/Name	When Query Applies	Query Description/Comments
**1)** Identify all visits/CRF's that have been submitted for review. (☑) Done by coordinating center, KTA and EDC.	View in OpenClinica Home or Subject Matrix	All Visits	Select the required sites to get an idea of how much work you may have at a given time. Using an "as of" date (type in as 01-Jan-12, for example) will identify all visits that have reached the milestone from the date specified to the current date. In order to see ALL visits since the beginning of the study that have been submitted for review, do not enter an "as of" date. You can also use the statistics provided to you upon log on to the system on the Home page
**Demographics, Vital Signs and Consent**			
**The above will be checked by the randomization system in Edinburgh during initial data entry**			
**Medical History**			
**The above will be checked by the randomization system in Edinburgh during initial data entry**			
**2)** Baseline visit all required	BV001	Check NDR	Check Notes & Discrepancies for notes
**3)** Discharge (Utskrivning)	UV001	Run query on dead patients	List patients who have died without a death date before being discharged
**4)** Discharge (Utskrivning)	UV002	Run query to check date of first dose	Lists patients where date of first dose has not been entered and if a reason is given .
**5)** Discharge (Utskrivning)	UV003	Run query to check last dose	If test drug was discontinued before discharge check that date for last dose and the reason test drug was continued
**6)** Week 1 visit	V1001	Check datum	List patients with no contact date and that a reason is given. Check also that an AE/SAE or

Question	Query Tool/Name	When Query Applies	Query Description/Comments
			AVLIDEN CRF have not been completed
**7)** Week 1 visit	V1002	Check if stopped	List patients who have stopped taking the test drug and that reason and date was entered
**8)** Month 1	M1001	Check datum	List patients with no contact date and that a reason is given
**9)** Month 1	M1002	Check if stopped	List patients who have stopped taking the test drug and that reason and date was entered
**10)** Month 3	M3001	Check datum	List patients with no contact date and that a reason is given
**11)** Month 3	M3002	Check if stopped	List patients who have stopped taking the test drug and that reason and date was entered
**12)** Month 6	M6001	Check datum	List patients with no contact date and that a reason is given
**13)** Month 6	M6002	Check if stopped	List patients who have stopped taking the test drug and that reason and date was entered
**14)** Month 6	M6003	Check if hospitalized	Check and validate if an SAE has been created
**15)** Month 7	M7001	Check if interview done	Check that interview was done if > 8 months have been passed since study start
**16)** Month 7	M7001	Check if hospitalized	Check and validate if an SAE has been created if hospitalization occurred within the seven month study period according to the AE/SAE manual.
**17)** Month 12	M12001	Check for SAE	Check for SAE during the 7 months follow up period
**18)** Month 12	M12002	Check if died	Check if patient died during follow-up period and if yes that the CRF AVLIDEN has been completed

**ADVERSE EVENT**			
**19)** AE/SAE CRF	AE001	Run on all patients with an AE	Verify that for all AE/SAE all the fields have been completed or commented on.
**20)** AE/SAE CRF	AE002	Run on all patients with AE	Verify where outcome is death that the CRF AVLIDEN has been completed.
**21)** Identify all missing information in the AE module.	AE004	All Visits	Run on all AE/SAE CRF's
