# Supplementary material for: Update on the EFFECTS study of fluoxetine for stroke recovery: a randomised controlled trial in Sweden
Source: Trials. 2020 Feb 28;21:233. doi: 10.1186/s13063-020-4124-7 (PMC7048055; doi:10.1186/s13063-020-4124-7)
Supplement: Supplementary file 10 — Additional file 10. EFFECTS latest DMC charter. [file 13063_2020_4124_MOESM10_ESM.pdf]

EFFECTS

Efficacy oF Fluoxetine – a randomisEd Controlled Trial in Stroke

**Independent Data Monitoring Committee (DMC)
CHARTER**

Trial Identifiers

Sponsors	Karolinska Institutet .<br>Representative for sponsor: Erik Lundström, MD, PhD,
Funders	Swedish Research Council, Swedish Stroke Association, Swedish Heart-Lung Foundation, King Gustaf V:s and Queen Victoria's Freemason Foundation
Chief Investigator	Dr Erik Lundström
EudraCT Number	2011-006130-16
Swedish Medical Products Agency approval:	Date: 2014-08-08.<br>Official Records No. 5.1-2014-43006)
Approval of Regional Ethical Committee:	Date: 2013-09-30:<br>Official Record No: 2013/1265-31/2
ISRCTN Number	ISRCTN13020412
Version of DMC Charter	Version 7.2, date: 2015-11-19
Protocol Number	EFFECTS2012

DMC Members:

- Professor em Kjell Asplund, Chair
- Professor Andreas Terént
- Statistician Anders Ljungström

Unblinded Statistician: Anders Ljungström

DMC Coordinator: TBD

Approval signatures

The following individuals, by providing their signatures, indicate their understanding of and willingness to comply with the roles and responsibilities assigned to them in this Charter.

	Role	Date	Signature
Prof Kjell Asplund	Chair, DMC	2015-11-19	[signature]
Prof Andreas Terént	DMC member	2015-11-19	[signature]
Anders Ljungström	Unblinded Statistician, DMC Member	2015-11-19	[signature]
Dr Erik Lundström	Chief Investigator	2015-11-19	[signature]
Prof Katharina Stibrant Sunnerhagen	Chair, Steering committee	19/11 2015	[signature]

I Scope of EFFECTS DMC Charter

The EFFECTS Independent Data Monitoring Committee (DMC) will independently monitor patient safety and efficacy information, and study conduct, during the period of the trial.

The objective of the EFFECTS DMC Charter is to outline the specific purposes and functions of the DMC and those supporting its activities, and the procedures for data abstraction and data delivery to and from the DMC members for review purposes.

The CHARTER for EFFECTS has been developed in direct collaboration with the UK-based FOCUS trial.

II Composition of the EFFECTS DMC

The DMC will comprise three (3) members. The DMC members will include two physicians with stroke expertise as well as a Biostatistician with clinical trial and prior DMC experience. The Sponsor, Karolinska Institutet/Erik Lundström (Chief Investigator) and the Steering Committee will approve all DMC members.

DMC members will not be involved as principal investigators or subinvestigators in the EFFECTS study. In addition, DMC members must not have a conflict of interest that would bias their review of trial data (e.g. DMC members must not have a financial interest that could be substantially affected by the outcome of the study, strong views on the relative merits of the study drug, relationships with individuals in trial leadership positions that could be considered reasonably likely to affect their objectivity, or involvement in any potential competing trial).

The Unblinded Statistician, a member of the DMC, will generate data and reports for the DMC to review.

All DMC members are expected to serve from study start until the study is completed, as defined by final database lock. Should it be necessary for a member to resign, the member must submit the effective date of resignation in writing to the Sponsor, Steering Committee Chair, DMC Chair, and Chief Investigator. In the event a member resigns, the Steering Committee Chair, DMC Chairman and Chief Investigator, will initiate the process to identify a replacement member.

III DMC Contacts and *ad hoc* Consultants

DMC contacts and *ad hoc* consultants are not considered to be members of the DMC. The official DMC contacts are named on the DMC roster and will be appointed as follows:

The Chief Investigator will assign a DMC Coordinator who will provide administrative, logistical, and coordinating services to the DMC. The DMC Coordinator will serve as the primary, administrative point of contact for communications with the DMC members and DMC-related issues and will liaise with the Steering committee and the operational leads on the project team, as appropriate.

The Chief Investigator will serve as a primary contact person for the DMC and DMC issues.

The DMC may, with prior approval from the Steering Committee Chair, contact and involve selected expert consultants who may, in strict confidence, provide additional, relevant insight or expertise to the DMC, regarding any specific issues that may arise.

As a rule, DMC contacts and consultants must not attend closed sessions of DMC Data Review Meetings.

The DMC Chairman will ensure that DMC contacts and consultants are not inappropriately exposed to unblinded data made available to the DMC.

IV EFFECTS DMC responsibilities

The EFFECTS DMC is an independent expert advisory group commissioned and charged with the responsibility of evaluating cumulative safety, efficacy and other clinical trial data at regular intervals. As such, the primary objective of the DMC is to monitor the safety of the subjects in the EFFECTS study by reviewing the available clinical data at scheduled time points including at least yearly meetings (which may be face to face or via teleconference) and on an *ad hoc* basis as needed.

After the review of each Data Report has been completed, the DMC Chair will provide the official DMC recommendation to the Sponsor, Chief Investigator and to the Chair of the EFFECTS Steering Committee regarding the appropriateness of continuing the study, from a safety and efficacy perspective, as well as any other recommendations relevant to study conduct and/or patient safety.

Specifically, the DMC members are authorised and expected to perform the following functions:

- Safeguard the interests of trial participants.
- Provide approval for and operate in accordance with the specifications outlined in this DMC Charter.
- Monitor the safety and efficacy of the trial intervention, through scheduled review of accumulating clinical data from the ongoing clinical trial and taking into account information from external sources.
- Consider the need for additional unscheduled reviews of study data.
- Review and evaluate the content of all unblinded Data Reports received.
- Ensure the confidentiality of all information received relating to the trial.
- In the event of further funding being required, to provide to the EFFECTS Steering Committee and funder(s) appropriate information and advice on the data gathered to date in a manner that will as far as possible protect the integrity of the study.
- Participate in and vote on DMC recommendations bearing in mind the fact that ethical considerations are of prime importance.
- Make clear recommendations to the EFFECTS Steering Committee, with the Steering committee chair as the principal contact.

The DMC will review safety outcomes, including serious adverse events. Review of safety data should occur after 150, 300, 600, 900 and 1200 patients 6-month follow-up data. No formal boundaries will be used for terminating the study for safety reasons but clear and consistent evidence of net harm that overrides any benefit should be apparent.

A formal interim analysis to assess efficacy will occur when approximately 67% of the planned primary efficacy events have accrued. The DMC may recommend early termination of the trial for overwhelming superiority of fluoxetin over control. A modified Haybittle-Peto monitoring boundary will be used as a guideline. If the primary efficacy comparison exceed 4 standard

errors in value, the DMC will initiate another interim analysis to be performed a minimum of 3 months later. If the monitoring boundary remains crossed, the DMC may recommend that the trial be terminated early for overwhelming superior efficacy of fluoxetine. No adjustment of the significance level for the final analysis is required.

The DMC will not be asked to make any recommendations of whether the trial should be stopped on the basis of futility, i.e. that the trial if it recruits to its targets sample size is unlikely to demonstrate a benefit from the trial fluoxetine.

Throughout the trial, the DMC Chair will take responsibility for the Committee's operation and will be authorised and charged with the following responsibilities:

- Chair DMC Data Review meetings.
- Ensure that all relevant data have been reviewed by the DMC members and that all issues have been addressed.
- Ensure that blinded individuals (i.e. the DMC Coordinator, DMC contacts, and DMC consultants) are not inappropriately exposed to confidential and/or unblinded data.
- Ensure that only the members of the DMC are present during DMC deliberations, when DMC recommendations are discussed and DMC voting procedures are conducted.
- Ensure the generation of confidential, written minutes of all closed sessions of any DMC Meetings and maintain these minutes as confidential to DMC members only, until the final (end of study) database lock is complete.
- Ensure DMC approval of minutes of open and final sessions of all DMC meetings.
- Communicate, author, sign, and provide the official, final recommendations of the DMC within specified timelines and according to the specifications outlined in this charter. If the DMC is divided in opinion on any major issue affecting the DMC's recommendation to the Sponsor and EFFECTS Steering Committee, the DMC Chair is responsible for assembling and presenting the majority and dissenting opinions for all recommendations considered.
- Arrange for consultation(s) and/or request additional data, as deemed necessary.
- If deemed appropriate by the DMC, at appropriate intervals, arrange a teleconference meeting with the Chairs of the DMC committees for the FOCUS (Professor Peter Langhorne) and AFFINITY (Professor Robert Herbert, Australia) trials. If necessary, to discuss accumulating data in strict confidence and any implications for the continuation of each of the trials. Each Chair may then need subsequently to consider whether to arrange a meeting of their respective trial DCM to discuss any issues that may arise from this liaison group.
- Maintain a secure central file of all data outputs received for DMC review and all minutes of all sessions of DMC meetings. Provide a copy of this file to the Sponsor, through the Chief Investigator, once the final (end of study) database lock is complete.

V Steering committee responsibilities

The Steering committee (in which the Chief investigator is a member, and in which the Chief investigator concurrently also represents the sponsor) will have the following responsibilities with respect to the EFFECTS DMC:

- Provide final approval of the DMC Chairman and Members to serve on the DMC.
- Assign a DMC Coordinator who will provide administrative, logistical, and coordinating services to the DMC.
- Ensure that the DMC Unblinded Statistician has access to data in the Clinical Data Management (CDM) system and information on allocation group, as requested by the DMC. The oblindande statistician will be provided data via CDM system after 150, 300, 600, 900 and 1200 patients. The allocation groups are not stored in the main OpenClinica database. The allocation code list must only be maintained by the DMC unlinded statistician
- Ensure relevant external clinical or other data on the safety of study interventions are provided to the DCM.
- Ensure that DCM members are informed of trial progress and issues at least every 12 months.
- In preparation for data review meetings, ensure that the DCM receive a general summary of the status of the trial and any relevant clinical issues.
- Attend all open and final sessions of DCM meetings, as needed.
- Arrange for fair and reasonable reimbursement to DCM members for their data monitoring activities (any study-related travel costs, such as transportation, lodging, and meals).
- Maintain ultimate responsibility for safe study conduct.

VI Unblinded Statistician responsibilities

The responsibilities of the Unblinded Statistician are as follows:

- Provide approval for and operate in accordance with the specifications outlined in this DMC Charter.
- Work with other DMC members to determine the data that are necessary for the DMC Data Reports.
- Create and execute computer programmes to generate the DMC Data Report and transfer those reports to DMC members in a secure and confidential manner.
- A copy of the treatment code will be kept by the DCM statistician The DMC can, at any time, in addition to the scheduled safety analyses at 150, 300, 600, 900 and 1200 patients 6-month follow-up data and the interim analysis request certain specified data from database to aid them in their function to maximize patient safety". The DMC unblinded statistician will define what data will be needed at each timepoint. The data will be provided to the DMC unblinded statistician in an encrypted coded ZIP file.
- Ensure that the content of unblinded study reports or details of discussions at DMC meetings are treated in the strictest confidence and are not revealed to any non-DMC member prior to study closedown, without the written approval of the DMC Chairman.

- Maintain a secure and confidential archive of electronic copies of datasets and related programs provided to the DMC Biostatistician.

VII DMC Coordinator responsibilities

The DMC Coordinator will provide administrative, logistical and coordinating support to the DMC members. The DMC Coordinator will be charged with the following responsibilities:

- Provide approval for and operate in accordance with the specifications outlined in this DMC Charter.
- Serve as the primary, administrative point of contact for the DMC members and as the main liaison between the EFFECTS operations teams and the DMC members.
- Coordinate the implementation of the schedule for preparation and distribution of Data Reports to DMC members.
- Follow-up to verify that all data required by the DMC is provided according to an agreed timeframe.
- Coordinate arrangements for all data review meetings and DMC ad hoc meetings, as outlined in this charter.
- Receive and arrange payment of DMC member invoices and expense reports, e.g. for travel to/from DMC meetings

VIII DMC Member involvement in protocol review and training

All DMC members will have the opportunity to review and comment on any proposed amendments to the protocol. The Chief Investigator will respond to all queries from the DMC on details of proposed amendments.

IX DMC Data Reports

DMC members will receive all DMC Data Reports directly from the Unblinded Statistician, who will receive information on randomization group at least four weeks prior to a scheduled data review meeting.

DMC Data Reports will be provided to the DMC members at least one week prior to scheduled data review meetings.

Data included in each DMC Data Report will be cumulative-to-date at the time of the established data cut-off. The cut-off date for the data included in the Data Reports, as well as the current enrolment figures, will be stated in the report.

The DMC may request additional information on individual patients, as needed.

Data Reports for review by the DMC will be presented on a Group A, Group B basis. The DMC members will be informed separately of the true treatment assignments associated with the groups.

During the period of recruitment into the study, the Unblinded Statistician will perform interim analyses on major outcome events (including efficacy, safety and serious adverse events) along

with any other analyses that the committee may request.

In the context of EFFECTS, the balance between safety and efficacy should be considered.

With respect to safety and efficacy the following outcomes in particular will initiate discussion and minuting of detailed reasons for recommending early stopping or continuation of the study:

- Dependency: modified Rankin Scale (mRS);

Serious adverse events (SAEs), in particular

- all cause mortality
- stroke/TIA
- myocardial infarction
- upper gastrointestinal bleeding
- serious falls
- serious fractures
- epilepsy/seizures
- attempted suicide/self harm
- hyponatraemia
- neuroleptic malignant syndrome-like events
- anaphylactoid reactions
- serotonergic syndrome
- pancytopenia, thrombocytopenia or haemolytic anaemia

In making any decision, the committee will consider the overall internal and external evidence, the multiplicity of testing and the possibility that the trends in the data might be reversed with longer follow-up or increased recruitment.

In the light of these analyses, the DMC will advise the Chair of the EFFECTS Steering Committee and Sponsor (via the Chief Investigator) if, in their view, the randomised comparisons in EFFECTS have provided both (i) "proof beyond reasonable doubt" that for all, or for some, specific types of patient, treatment is clearly indicated or clearly contraindicated, and (ii) evidence that might reasonably be expected to influence materially the patient management of the many clinicians who are already aware of the results of any other relevant trials.(DAMOCLES study group 2005; Grant, Altman et al. 2005). Appropriate criteria of proof beyond reasonable doubt cannot be specified precisely, but the DMC will work on the principle that a difference of at least 3 standard errors in an interim analysis of a major outcome event (e.g. death from all causes or independent survival at six months) may be needed to justify halting, or modifying, a study before the planned completed recruitment. This criterion has the practical advantage that the exact number of interim analyses would be of little importance, and so no fixed schedule is proposed.

Following a report from the DMC, the Trial Steering Committee will decide whether to modify entry to the study (or seek extra data). Unless this happens however, the Steering Committee, the collaborators and central administrative staff will remain ignorant of the interim results.

X DMC Committee meetings

The committee will convene mainly via telephone conferences which should take place as soon as reasonably possible after the committee members have received data from the trial statistician; discussions must include all three members. Meetings should take place at least 12 monthly, or more frequently if necessary. The DMC Coordinator will organize the meetings but not otherwise participate.

The meetings will commence with an 'open' session which will also be attended by the Chair of the

Steering committee and the Chief Investigator (or representative) who will give an update on the trial's status. This will be followed by the 'closed' session attended by DMC members only. 'Open' session minutes will be taken by the DMC coordinator and circulated for approval and 'Closed' minutes and recommendation will be drafted by the DMC Chair and agreed by the DMC members. The DMC Chairman will report to the Chief Investigator.

XI DMC Data

Data tables, listings and graphical displays will be reported as appropriate for the whole trial, and for fluoxetine group and placebo group based on the following data. The DMC will receive a mock-up of the Report for approval prior to the first meeting at which data will be reviewed. Data will be listed by Groups A & B, (in case the report becomes 'lost') where A, B, stand for respectively fluoxetine and placebo.

Trial status:

- Timeline for trial.
- Number of patients randomized.
- Cumulative recruitment graph.
- Discontinuation data including reasons for discontinuation.
- Completeness of data for baseline (day 0), discharge, 1 month, 6 months and 12 months.

Baseline (pre-randomisation) data:

- Demographic: Age; sex; ethnic group.
- Stroke management:
  - Inpatient vs outpatient at time of randomization
  - time from stroke to randomization;
- Predicted outcomes based on Counsel model
- Motor deficits
- Aphasia
- Depression (based on PDQ 2/DSM-IV)

Discharge (for inpatients) data (local centre):

- Number of patients with data.
- Safety: PI will check all AEs in their patients but those to be reported are Serious adverse events (SAEs).
- Adherence with trial medication

1 month data (over the telephone, local centre):

- Number of patients with data.
- Safety: PI will check all AEs in their patients but those to be reported are Serious adverse events (SAEs)
- Adherence with trial medication

3 month data (over the telephone, local centre, prior to dispensing 2nd bottle of trial medication)

- Depression according to DSM-IV and MADRS
- Safety: PI will check all AEs in their patients but those to be reported are Serious adverse events (SAEs)

6 months data:

- Number of patients with data.
- Safety: PI will check all AEs in their patients but those to be reported are Serious adverse events (SAEs)
- Safety: Concomitant treatment with drugs known to interact with fluoxetin
- Adherence with trial medication
- *Primary outcome* modified Rankin Scale (mRS);

12 *months data:*

- Number of patients with data.
- Safety: PI will check all AEs in their patients but those to be reported are Serious adverse events (SAEs)
- Safety: Concomitant treatment with drugs known to interact with fluoxetin
- Adherence with trial medication

Primary outcome modified Rankin Scale (mRS);

XII Records Retention

The DMC Chair will ensure a copy of the DMC file (i.e., copies of all reports reviewed by the DMC and copies of final minutes of all sessions of any DMC meeting) is sent to the Chief Investigator after the end of the study. It will be the responsibility of the Chief Investigator, on behalf of the Sponsor, to arrange for long-term archiving.

XIII Indemnification and Liability

The Sponsor shall indemnify, defend and hold harmless each DMC member (and their employer where their DMC member duties are undertaken in the course of their employment), from and against any and all losses, damages, liabilities, reasonable attorney fees, court costs, and expenses (collectively "Losses") resulting or arising from any third-party claims, actions, proceedings, investigations or litigation relating to or arising from or in connection with the performance of responsibilities by such DMC member contemplated herein, except to the extent any such Losses have resulted from a breach of such DMC member's obligations hereunder or from any wilful or intentional misconduct of the DMC member seeking indemnity hereunder.

References

DAMOCLES study group (2005). "A proposed charger for clinical trial data monitoring committees: helping them to do their job well." Lancet **365**: 711-722.

Grant, A. M., D. Altman, G,, et al. (2005). "Issues in data monitoring and interim analysis of trials." Health Technology Assessment **9**(7): 1-237.

Appendix 1: Contact details

DMC members:

Professor em Kjell Asplund (Chair)
Reimersholmsgatan 59
117 40 Stockholm
Tel +46 8 669 5175
mobile phone +46 70 573 2281
e-mail kjellasplund1@gmail.com

Professor Andreas Terént (Member)
tel +46 18 611 2752 (Akademiska)
mobile phone +46 70 345 2003
e-mail andreas.terent@akademiska.se

Anders Ljungström (Member, Unblided Statistician)
mobile phone + 46 70 759 0260
-email anders.ljungstrom@tele2.se

Chief Investigator: *Dr Erik Lundström,*

Sponsor: *Karolinska Institutet/Erik Lundström*

Sponsor's representative: *Erik Lundström, erik.lundstrom@gmail.com,* EFFECTS 24-timmars helpline 073 66 37 444

Chair DMC FOCUS:
Professor Peter Langhorne
Professor of Stroke Care (Institute of Cardiovascular and Medical Sciences)
R 2.43, Level 2, New Lister Building, Glasgow Royal Infirmary, Glasgow G31 2ER
telephone: 0044 1412018510
email: Peter.Langhorne@glasgow.ac.uk

Chair DMC AFFINITY:
Professor Rob Herbert
Senior Principal Research Fellow
Neuroscience Research Australia
www.NeuRA.edu.au
Barker Street Randwick Sydney NSW 2031 Australia
PO Box 1165 Randwick Sydney NSW 2031 Australia
Email: r.herbert@neura.edu.au
T +61 2 9399 1833 F +61 2 9399 1082

To be discussed (TBD): Any further person in any position
